# Vanishing Quantum Confinement Enables Bright and Thermally Excited Multicarrier Emission from Semiconductor Nanocrystals

**DOI:** 10.1021/acsnano.5c19908

**Published:** 2026-01-20

**Authors:** Tjom Arens, Sander J.W. Vonk, A. Willem Vlasblom, Margarita Samoli, Daniel Vanmaekelbergh, Pieter Geiregat, Zeger Hens, Freddy T. Rabouw

**Affiliations:** 1 Soft Condensed Matter & Biophysics, Debye Institute for Nanomaterials Science, 8125 Utrecht University, Princetonplein 1, Utrecht 3584CC, The Netherlands; 2 Condensed Matter & Interfaces, Debye Institute for Nanomaterials Science, 8125 Utrecht University, Princetonplein 1, Utrecht 3584CC, The Netherlands; 3 Optical Materials Engineering Laboratory, Department of Mechanical and Process Engineering, ETH Zurich, Zurich 8092, Switzerland; 4 Physics and Chemistry of Nanostructures, Department of Chemistry, Gent University, Krijgslaan 281, Gent 9000, Belgium; 5 NOLIMITS, Core Facility for Non-Linear Microscopy and Spectroscopy, Gent University, Krijgslaan 281, Gent 9000, Belgium

**Keywords:** single-particle spectroscopy, multicarrier excited states, suppressed Auger recombination, weak quantum confinement, temperature-dependent spectroscopy

## Abstract

Recently, nanocrystals in the regime of vanishing quantum confinement—termed bulk nanocrystals (BNCs)—have demonstrated optical gain characteristics. While their high-power lasing performance was demonstrated convincingly, the photophysics at low and intermediate powers—where charge-carrier populations are discrete—remain unexplored. Using single-photon avalanche diode (SPAD) array technology, we resolve the dynamics and energetics of six multicarrier excited states in individual CdSe/CdS core/shell BNCs, containing up to four electrons and two holes. Each state exhibits bimodal emission, indicative of thermal equilibrium between closely spaced electron and hole levels, confirmed via temperature-dependent single-particle measurements. Quantification of radiative and nonradiative decay channels reveals strongly suppressed Auger recombination through both the negative- and positive-trion pathways. We present a model that combines statistical scaling of rate constants with Fermi–Dirac thermal occupations of electron and hole levels, bridging the transitional regime between quantum-confined and bulk nanocrystals, and providing a comprehensive framework for understanding this emerging class of materials.

## Graphical abstract

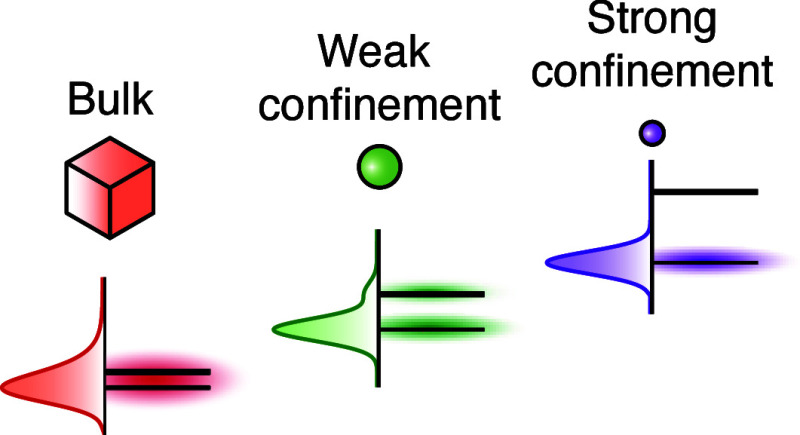

## Introduction

Semiconductor nanocrystals (NCs) are used as light-emitting components for commercial displays and hold great promise for lighting and laser applications.^1^ Over the past three decades, design strategies such as core–shell architectures^2,3^ and alloyed interfaces^4–6^ have been optimized to enhance photoluminescence efficiencies by mitigating surface-related losses and suppressing nonradiative Auger recombination. Auger processes, which quench multicarrier excited states under strong excitation, have long posed a key limitation for achieving efficient and sustained lasing in NCs, restricting early demonstrations to short-pulse excitation and high thresholds.^7^ These challenges were partially overcome by selectively delocalizing carriers into the shell^8,9^ or by grading the confinement potential at the core–shell interface.^4–6^

More recently, attention has shifted from shell engineering to the effect of core size. Large NCs with diameters comparable to or exceeding the Bohr radius have demonstrated efficient spontaneous and stimulated emission from multicarrier states, indicative of strongly suppressed Auger recombination.^10–13^ Depending on composition, such particles are termed quantum shells^14,15^ if the innermost core is a high-bandgap material, or bulk nanocrystals^11,12^ (BNCs) if the core is the material with the lowest bandgap. These materials show long gain lifetimes, consistent with their ability to sustain optical gain under quasi-continuous-wave excitation.^11,12,16^ The lasing behavior and the gain spectra of BNCs were described using a bulk semiconductor model with continuous charge-carrier densities and a continuous density of energy levels.^17–19^ Yet, the structured gain spectra hinted at discrete energy levels.^11^ Moreover, charge-carrier densities are discrete around or below the gain threshold, which is the regime most relevant to lighting or laser operation. The behavior of NCs with vanishing quantum confinement, where the energy levels are dense but discrete and where charge-carrier densities are low but discrete, remains unexplored.

Addressing this challenge requires methods capable of resolving discrete multicarrier states in individual NCs. Ensemble transient-absorption studies have provided valuable insights,^20^ but quantitative interpretation is complicated by particle-to-particle variations in energetics and dynamics, as well as spontaneous charging^21^ and the simultaneous generation of a variety of multicarrier states. Single-particle spectroscopy can overcome these limitations by directly resolving multicarrier states,^19,22–24^ including charged configurations that arise spontaneously^25^ or can be introduced intentionally^26,27^ in laser design strategies.

In this work, we isolate and characterize the energetics and dynamics of six multicarrier states in Cd chalcogenide BNCs with an average core diameter of 10.5 nm, placing them in the transitional regime between bulk and quantum-confined. We resolve multicarrier states in individual BNCs with a number of holes up to 2 and electrons up to 4, using a method termed cascade spectroscopy^19,28^ or heralded spectroscopy^23,24^ on a single-photon avalanche diode (SPAD) array detector. The photoluminescence (PL) spectra are bimodal, with a relative intensity scaling with the number of charge carriers, highlighting thermal occupation of higher electron and hole levels. The expected temperature dependence of level occupations is confirmed with temperature-dependent single-particle measurements using specialized microscope substrates capable of localized heating.^29^ We quantify radiative and nonradiative decay pathways of each multicarrier state. Nonradiative Auger losses are spectacularly reduced compared to smaller NCs, as highlighted by the emission efficiency as high as 60% for a highly excited state with 2 holes and 4 electrons. Finally, we develop a unifying model of discrete electron and hole levels with Fermi–Dirac occupation statistics to describe the size regime between quantum confinement and bulk.

## Results and Discussion

### Resolving the Emission Spectra of Different Excited States in a Single Nanocrystal

We prepare our sample by spin-coating a strongly diluted solution of CdSe/CdS core/shell BNCs (core diameter: 10.1 ± 1.2 nm; total diameter: 15.4 ± 1.5 nm; mean ± standard deviation as determined from electron microscopy; Figure S1; cf. Bohr diameter: 6.8 nm^30^) with an ensemble quantum yield of approximately 20%^11^ onto a glass coverslip. The coverslip is fixed onto a microscope slide under a nitrogen atmosphere using an airtight adhesive spacer. The exclusion of oxygen stabilizes excess electrons in the conduction band and facilitates negatively charged multicarrier states.^31^ Indeed, the introduction of oxygen makes the BNCs less prone to charging (Figure S2). We perform pulsed-excitation single-BNC experiments with a 405 nm diode laser operating at a repetition rate of 1 MHz and a fluence corresponding to an average number of excitations per pulse of μ ∼ 0.2. The emission from a single BNC is spectrally dispersed by a transmission grating onto a one-dimensional SPAD array, which together act as a spectrometer setup with single-photon time-tagging capabilities and a spectral resolution of 1.84 nm (Figure 1a and Figure S3).^23,24^ We verify the presence of only one active emitter in the laser focus using a time-gating method (Supporting Information, Section S4).

**Figure 1:**
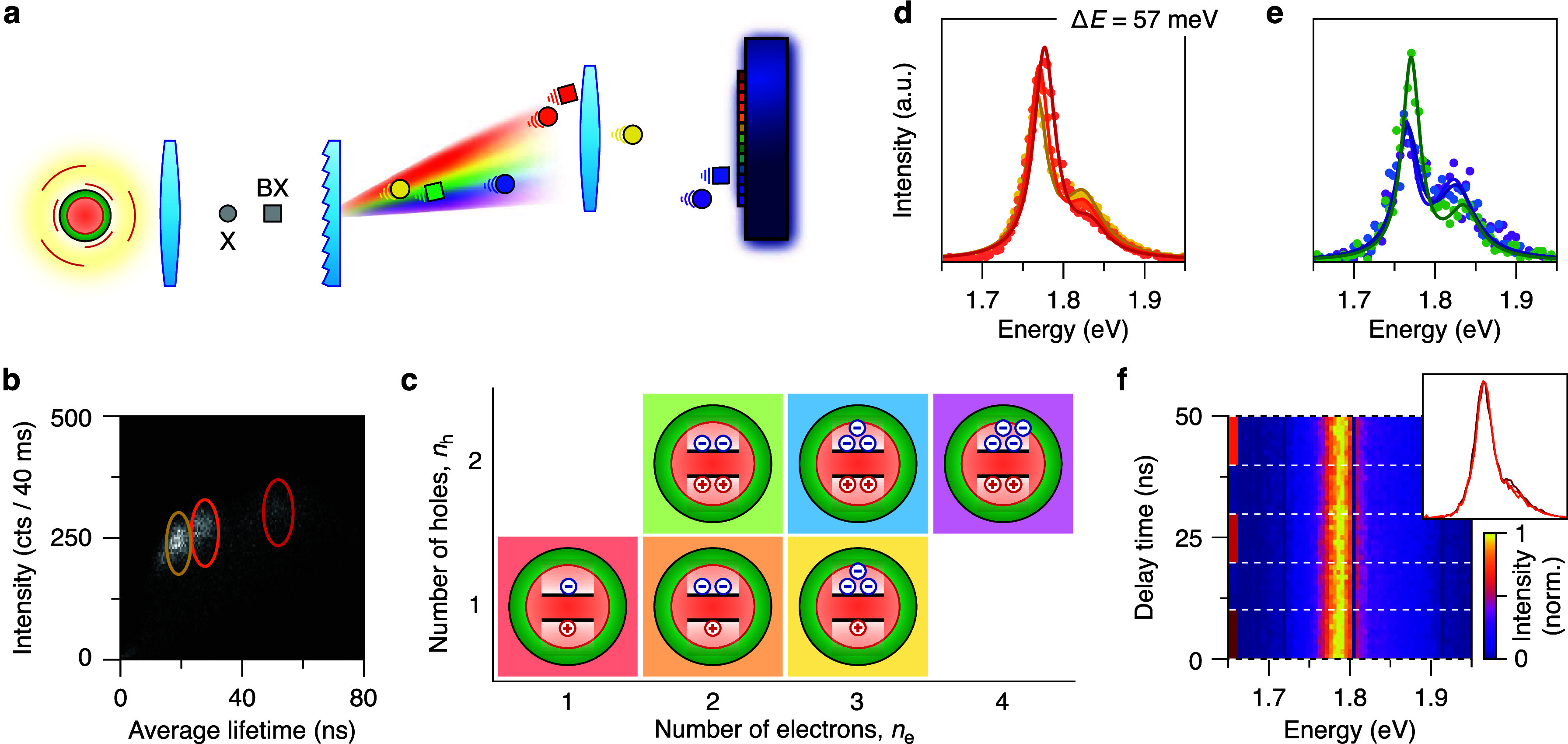
Experimental setup and emission dynamics of a single bulk nanocrystal. (a) Schematic of the single bulk nanocrystal (BNC) experiment. A 1 MHz pulsed laser excites an individual BNC, which subsequently emits exciton (circle) and biexciton (square) photons. The emitted photons are spectrally dispersed using a diffraction grating and imaged onto a linear single-photon avalanche diode (SPAD) array. Since biexciton and exciton photons are emitted sequentially, biexciton photons can be isolated by postselecting the first photon of each photon pair following a single excitation pulse. (b) Fluorescence-lifetime intensity distribution (FLID), showing fluctuations in average lifetime and intensity over a total experiment, caused by spontaneous charging and discharging of the BNC. Three distinct emissive states are identified based on their average lifetime: neutral (red), singly negatively charged (orange), and doubly negatively charged (yellow). (c) Combining the postselection of biexciton photons with charge-state identification enables the isolation of six distinct excited states, corresponding to configurations with one or two holes and up to four electrons. (d) Reconstructed photoluminescence (PL) spectra of the neutral exciton (red), singly charged exciton (= trion; orange), and doubly charged exciton (yellow). (e) Same as panel d but for the neutral biexciton (green), singly charged biexciton (blue), and doubly charged biexciton (purple). The rise in the relative intensity of the higher-energy peak with the number of excess electrons is attributed to increased population of the electron P level. (f) Normalized time-resolved emission map showing spectral evolution of the neutral exciton emission as a function of delay time. The inset displays the integrated spectra over delay ranges of 0–10, 20–30, and 40–50 ns (see Figure S8 for all exciton states with enlarged insets). At early times (0–10 ns), the higher-energy P-level contribution is enhanced, consistent with the presence of the neutral biexciton, which has a shorter lifetime than the exciton and contributes primarily at short delays. At later times, when only exciton emission remains, the spectral shape stabilizes. These results indicate that the two emitting levels are thermally coupled.

The emission intensity and excited-state lifetime fluctuate, caused by spontaneous charging and discharging of the BNC (Figure S5).^2,32^ We isolate the moments when the BNC is charged by analyzing the fluorescence-lifetime–intensity distribution (FLID; Supporting Information, Section S5.1), a 2D histogram that shows the number of photons in 40 ms time bins and the corresponding average fluorescence lifetime, as shown in Figure 1b. We distinguish three emissive states, which we ascribe to different degrees of negative charging of the BNC promoted by the oxygen-free atmosphere.^33^ Previous experiments with an external potential could promote such charging intentionally.^34^ We identify the highest-intensity state (300 cts/40 ms; 50 ns; red) as the neutral BNC and the two states with progressively lower intensity (265 and 240 cts/40 ms) and shorter lifetime (26 and 18 ns) as the singly (orange) and doubly (yellow) negatively charged states. The observation of bright charged states indicates that Auger quenching in these BNCs is weak. This behavior is consistent with the large particle size, which reduces Auger rates owing to volume scaling,^35,36^ and with the long gain lifetimes that point to suppressed Auger recombination.^11,12^

Our time-tagged spectral data allow the PL spectra of different multicarrier states to be reconstructed. Each photon recorded during the experiment is assigned to a different charge state based on the FLID analysis described above. Under low-excitation conditions, these photons originate mostly from singly (as opposed to doubly) excited states. Hence, the integrated spectra of the three charge states (Figure 1d) are those of the neutral exciton (1 electron + 1 hole), singly charged exciton (2 electrons + 1 hole), and doubly charged exciton (3 electrons + 1 hole). A fraction of the photons originates from doubly excited states, which we isolate from cascade events in our photon stream—more specifically, laser pulses followed by two photon-detection events. As the recorded signal originates from a single BNC, the first and second photon of a cascade must be emitted in succession, i.e. by decay from the doubly excited state via the singly excited state to the ground state (Supporting Information, Section S5.2).^19,23^ Figure 1e shows the PL spectra of the doubly excited states with different negative charges. We will refer to these as the neutral biexciton (2 electrons + 2 holes), singly charged biexciton (3 electrons + 2 holes), and doubly charged biexciton (4 electrons + 2 holes).

The spectra of Figure 1d,e are bimodal for all multicarrier states, with a dominant low-energy transition at 1.78 eV and a weaker high-energy transition at 1.83 eV. Visual inspection of the spectra shows a redshift of the emission energies for higher multicarrier states. Quantitative analysis (Figure S7) reveals a multicarrier binding energy of 2–6 meV per electron on the lower-energy emission peak, while the number of holes has no effect. Hence, the trion and biexciton binding energies are equal at 6 meV. The higher-energy emission peak redshifts along with the main peak (Figure S7). Notably, the intensity ratio between the two peaks changes: the relative intensity of the high-energy transition increases with the number of excess electrons for both the exciton and the biexciton emissions. These spectra are qualitatively different from room-temperature spectra of smaller quantum dots (QDs). Single-QD emission spectra sometimes show a tail on the red side of the main peak, but these are well understood as phonon replicas.^37,38^ In contrast, the side peak observed here is on the blue side.^11,12^

Asymmetric emission spectra have been observed in ensemble-scale studies of BNCs^10,11^ and quantum shells.^14^ The bimodal structure is more distinct in our single-BNC spectra because of reduced spectral broadening. Lv et al.^10^ ascribed the blue emission shoulder to excited particle-in-a-box electron and hole levels for their cubic BNCs. The equivalent in our spherical BNCs would be the P-symmetry levels. Indeed, the experimental energy splitting of Δ*E* = 57 meV is close to the expected energy difference between 1S_3/2_1S_e_ and the 1P_3/2_1P_e_ states of 82 meV from a simple single-carrier effective-mass model (eq S8). To check that the 1P_3/2_1P_e_ emission is due to thermal excitation of charge carriers—rather than transient emission as charge carriers thermalize—we construct a time-resolved emission map (Figure 1f for the neutral exciton; Figure S8 for all exciton states with enlarged insets). The constant spectral shape over delay times up to 50 ns is consistent with thermal coupling between excited states strongly outpacing recombination from either state.

The scaling of the intensity ratio between the two emission peaks with increasing number of charge carriers (Figure 1d,e) is as expected from the fermionic nature of charge carriers in BNCs. The degeneracy of the lowest 1S levels is low: *g*_e_ = 2 and *g*_h_ = 4 for the electron and hole, respectively. As the number of charge carriers increases, the thermal occupations of the 1S and 1P levels do not grow at the same pace. Instead, the fermion occupation of a level is limited by the degeneracy, as described by Fermi–Dirac statistics. Quantitative analysis of Fermi–Dirac occupations will follow later.

To confirm thermal occupation of P-symmetry energy levels we raise the temperature during single-particle spectroscopy. This is made possible with Interherence Vaheat coverslips^29^ with localized heating up to 100 °C through resistive heating of transparent conductive wiring (Figure 2a). We excite the sample in widefield and guide the emission from dozens of individual BNCs via a slit and a reflective grating onto a 2D electron-multiplying CCD camera (Figure 2b). To ensure characterization of the neutral exciton state (1 electron +1 hole), the experiments were conducted in air and under low-light continuous-wave excitation. We obtain values for the energy of the SS transition *E*_S_, the energy difference between the SS and PP transition Δ*E* and the P-to-S intensity ratio *R = I*_P_/*I*_S_, both at room temperature (20 °C) and at 60 °C by fitting Gaussians to the spectrum of each BNC (Supporting Information, Section S6).

**Figure 2:**
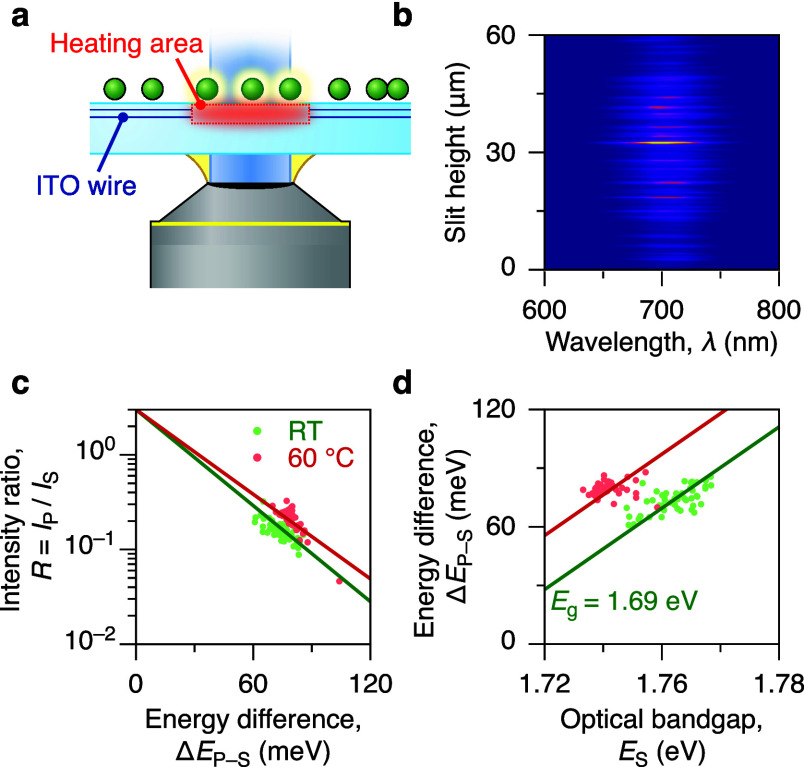
Temperature-dependent multiparticle spectroscopy. (a) Schematic of the experimental setup used for widefield, high-throughput temperature-controlled spectroscopy of single BNCs. Dozens of BNCs are excited simultaneously using widefield illumination through an oil-immersion objective, while an Interherence Vaheat substrate heats them. Emission is spectrally dispersed using a slit and grating and imaged onto an electron-multiplying CCD camera. (b) Time-averaged PL spectra of multiple single BNCs, which are the streaks at different heights on the camera image. Each BNC spectrum is fitted to extract the SS transition energy *E*_S_, the energy separation between the SS and PP transition Δ*E* and the P-to-S fluorescence intensity ratio *R* = *I*_P_/*I*_S_. (c) Scatter plot of the experimental *R* versus Δ*E* at room temperature (20 °C; green) and 60 °C (red). Each data point is an individual BNC. The intensity ratio increases for smaller Δ*E* and higher temperatures, consistent with thermally activated P emission. Solid lines are the expected intensity ratio as a function of Δ*E* according to a Boltzmann model with a degeneracy ratio of *g*_P_/*g*_S_ = 3. (d) Same as panel c but for the experimental Δ*E* versus *E*_S_. Solid lines are fits with a slope fixed by a particle-in-a-box model and the bulk bandgap as an optimizable parameter.

Variations in the intensity ratio between BNCs correlate with the energy splitting Δ*E* and increase at 60 °C compared to room temperature (Figure 2c). This is exactly as expected for thermal coupling between S and P levels. Unintentional variations between nominally identical BNCs in terms of particle size thus present a convenient test of thermal occupation. For the exciton state—with only a single carrier per band—the fermionic nature of charge carriers does not affect level occupations and Boltzmann statistics are expected. Indeed, the data points in Figure 2c match the Boltzmann model

$$R=\frac{g_{P}}{g_{S}}\exp\left(-\frac{\Delta E_{P-S}}{k_{B}T}\right)$$ 1

where *g*_P_/*g*_S_ = 3 is the degeneracy ratio between bright SS and PP exciton states (Supporting Information, Section S7.2), *k*_B_ the Boltzmann constant and *T* is the temperature (293 or 333 K). The good match of eq 1 to the data without any optimizable parameters indicate equal oscillator strength of SS and PP transitions.

We also observe a correlation between Δ*E*_P–S_ and *E*_S_ (Figure 2d). This is expected for energy variations due to size differences, as the particle-in-a-spherical-box model predicts a *V* ^–2/3^ scaling for both Δ*E*_P–S_ and *E*_S_ (Supporting Information, Section S7.3). Specifically, a relation of

$$\Delta E_{P-S}=\frac{\chi_{11}^{2}-\pi^{2}}{\pi^{2}}\left(E_{S}-E_{g}\right)$$ 2

is expected, where χ_11_ = 4.49 is the first zero of the spherical Bessel function of order 1 and *E*_g_ is the bulk bandgap energy of CdSe. We fit eq 2 to the data in Figure 2d, fixing the slope of the line and optimizing only *E*_g_. The fitted room-temperature value of *E*_g_ = 1.69 eV is slightly lower than literature values (1.74–1.76 eV),^11,39–41^ which can be understood from the 60-meV Stokes shift for this batch of BNCs (Figure S9). The fitted bandgap value at 60 °C is 1.67 eV. A decrease of the bandgap energy on the order of 10 meV between room temperature and 60 °C is consistent with expansion of the semiconductor lattice and increased electron–phonon interactions, as empirically described by the Varshni equation.^42^

### Characterizing the Intermediate Regime between Bulk and Quantum Confinement

Our observations lead to a conceptual understanding of the energetics of BNCs, which naturally links the description of charge carriers in strongly confined NCs and in a bulk semiconductor (Figure 3a–c). Strongly confined NCs have discrete energy levels and charge carriers occupy only the lowest level in the exciton ground state (Figure 3a). Higher energy levels are occupied only at high charge carrier densities or transiently following nonresonant excitation. On the other extreme of the size range, bulk materials have a continuous density of states and their occupation is described by Fermi–Dirac statistics (Figure 3c). The transitional regime—that of BNCs—is well described with a discrete-level model including thermal excitation of S electrons and holes to P levels (Figure 3b).

**Figure 3:**
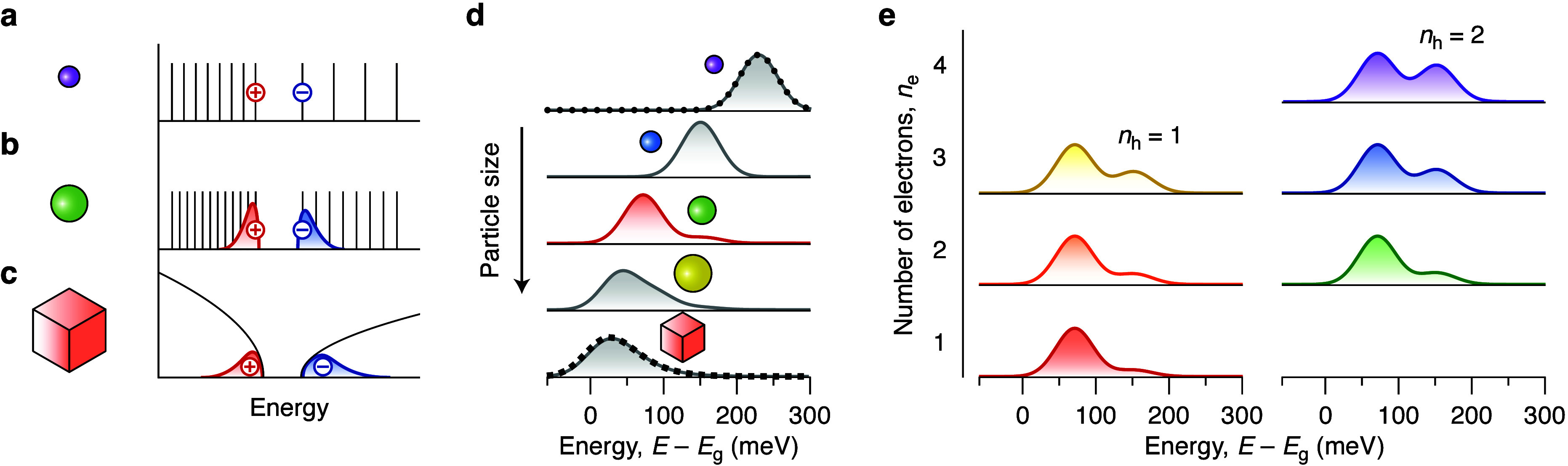
Energetics of multicarrier excited states in CdSe bulk nanocrystals. (a–c) Schematic representation of the energy-level separation and state filling across three particle size regimes: (a) strongly quantum-confined, (b) intermediate, and (c) bulk. In the intermediate regime, discrete energy levels remain, but their populations follow Fermi–Dirac statistics. (d) Model calculations for the emission of the neutral exciton state, based on the effective-mass model for energy level spacing and Boltzmann distribution for level occupation. Different graphs represent different NC sizes ranging from 3× smaller (purple) to 10× larger (red) than our BNCs. The dashed lines on the top and bottom spectra are the predictions of conventional models for strongly confined NCs and for a bulk semiconductor, respectively. Each calculated spectrum is broadened by 25 meV. Our model connects the two regimes of bulk and quantum-confined NCs. (e) Extension of the model to include multiple electrons and/or holes per NC, by incorporating the fermionic nature of the charge carriers. In agreement with experimental observations, the calculated spectra show an increase in the relative intensity of high-energy peaks with increasing electron number.

Figure 3d presents theoretical emission spectra of the neutral exciton state for increasing particle size, where each spectrum is broadened by 25 meV (Supporting Information, Section S7.4). These simple calculations neglect Coulomb interactions between carriers and assume equal oscillator strength for the 1S_3/2_1S_e_ and 1P_3/2_1P_e_ excitons. The calculated spectrum for the smallest particles (3× smaller than our BNCs) matches the prediction of a conventional model for strongly confined NCs (dashed black line). The calculated spectrum for the largest particles (10× larger than our BNCs) matches the prediction of a conventional bulk model (dashed black line). Our model reproduces the expected behavior in both limiting regimes and captures the emergence of discrete spectral features in the intermediate, transitional size range. Thermal excitation was thus the missing link for the description of the transitional size regime.

Describing thermal excitations for multicarrier states beyond the neutral exciton requires Fermi–Dirac statistics, accounting for the fermionic nature of electrons and holes. This leads to increased occupation of higher excited states at elevated charge-carrier densities (Supporting Information, Section S8). Consistent with experimental observations (Figure 1d,e), the calculated relative intensity of the high-energy peaks increases with the number of electrons in the NC (Figure 3e).

Thermal excitation is also expected to be reflected in the recombination dynamics. We quantify the recombination rates of the excited states identified in Figure 1. Specifically, we reconstruct the (cascaded) time-averaged decay curves of the exciton and biexciton for each charged state (Figure 4a,b). We find lifetimes (Supporting Information, Section S5.3) for the neutral, singly charged and doubly charged biexciton of τ_BX_ = 11.2 ns, τ_BX^–^_ = 7.5 ns, and τ_BX^2–^_ = 5.8 ns, respectively. We then extract the exciton lifetimes from a biexponential fit (Supporting Information, Section S5.4) to the total decay curves, fixing the short lifetime component at the biexciton lifetime. The lifetimes for the neutral, singly charged and doubly charged exciton are τ_X_ = 59 ns, τ_X^–^_ = 28 ns, and τ_X^2–^_ = 20 ns, respectively.

**Figure 4:**
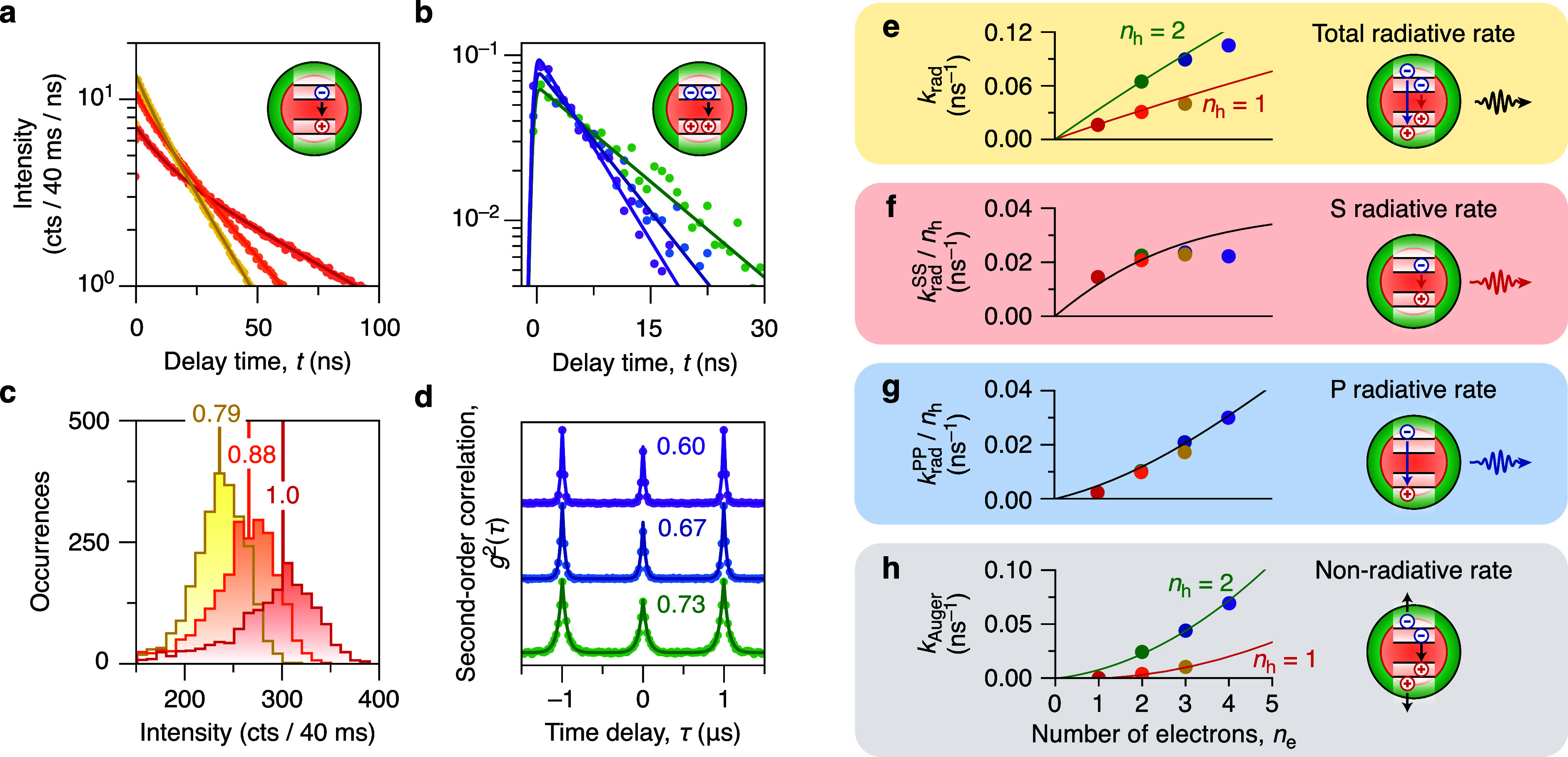
Statistical scaling validated over many excited states in a single BNC. (a) Photoluminescence decay curves of the neutral (red), singly charged (orange), and doubly charged (yellow) states. (Charged) Exciton lifetimes are extracted from the slow component of a biexponential fit, while the fast component is attributed to the (charged) biexciton. (b) Photoluminescence decay curves of the neutral (green), singly charged (blue), and doubly charged (purple) biexciton states, reconstructed by analyzing photon cascades. (c) Histogram of photon count rates from the neutral (red), singly charged (orange), and doubly charged (yellow) states, the solid lines show the center of a Gaussian fit. Assuming a unity quantum efficiency of the neutral exciton, we estimate the efficiencies of the other states from the relative count rates.^43^ (d) Second-order correlation functions of the neutral (green), singly charged (blue), and doubly charged (purple) states of the BNC. We estimate the (un)­charged biexciton quantum efficiencies from the peak height in the correlation function and the (un)­charged exciton quantum efficiencies determined in panel c. (e) Total radiative decay rate *k*_rad_ as a function of the number of electrons *n*_e_ in a multicarrier state. Solid lines: expected trends following eq 6 for states with *n*_h_ = 1 (red) and *n*_h_ = 2 (green), taking *B*′ such that the theoretical and experimental *k*_rad_ match for the biexciton. (f) SS radiative transition rate (eq 7) scaled with *n*_h_ and as a function of *n*_e_. Solid: expected trend according to eq 8, using *B*′ from panel e. (g) Same as panel f but for the PP radiative transition rate. (h) Total nonradiative decay rate as a function of *n*_e_. The solid lines are the expected Auger decay rates for the various charge configurations (eq 9) calculated using Auger coefficients *C* ^–^ = 0.0017 and *C* ^+^ = 0.0040 ns^–1^ for the negative and positive trion pathways, respectively. Figure S12 shows the minor effect that energy shifts due to multicarrier Coulomb interactions could have on the modeling results shown in panels e–g.

To distinguish radiative and nonradiative decay contributions, we first determine the quantum efficiencies of the excited states from their relative count rates and intensity-correlation analysis (Supporting Information, Section S5.5). The quantum efficiency of the neutral exciton is assumed to be 100%, because in single-BNC measurements we observe only the bright fraction of BNCs of the synthesis batch.^43^ The quantum efficiencies of the charged exciton states are obtained from the relative count rates compared to the neutral exciton (Figure 4c), giving η_X^–^_ = 88% for the singly charged exciton and η_X^2–^_ = 79% for the doubly charged exciton. Under low excitation fluences, the biexciton-to-exciton quantum efficiency ratio can be determined from the intensity-correlation function *g* ^(2)^ (Figure 4d):^44^

$$\frac{\eta_{\mathrm{BX}}}{\eta_{X}}=\frac{g^{\left(2\right)}\left(0\right)}{g^{\left(2\right)}\left(\pm T\right)}$$ 3

where *g* ^(2)^(0) is the amplitude of the zero-delay peak and *g* ^(2)^(±*T*) is the average amplitude of the side peaks. Accounting for the finite quantum efficiencies of charged excitons (Figure 4c), we determine the quantum efficiencies of the neutral, singly charged and doubly charged biexciton at η_BX_ = 73%, η_BX^–^_ = 67% and η_BX^2–^_ = 60%, respectively. These high quantum efficiency values of multicarrier states are consistent with suppressed Auger recombination in large NCs, but are exceptionally high here, exceeding 50% even for states with up to 6 charge carriers.^15,33,45^ We then convert the lifetimes and the quantum efficiencies into radiative *k*_*i*,r_ and nonradiative *k*_*i*,nr_ decay rates for each state *i:*

$$k_{i,\mathrm{rad}}=\frac{\eta_{i}}{\tau_{i}},\mathrm{and},k_{i,\mathrm{nonrad}}=\frac{1-\eta_{i}}{\tau_{i}}$$ 4

The radiative and nonradiative decay rates of six multicarrier states are an excellent data set to test the validity of statistical scaling on recombination rates and the possible influence of thermal occupation. In bulk, where energy levels are effectively degenerate and thermal effects are negligible, the radiative decay rate scales simply with the number of electrons *n*_e_ and holes *n*_h_:

$$k_{\mathrm{rad}}=Bn_{e}n_{h}$$ 5

In BNCs, where energy levels remain discrete, the scaling is determined by the expected number of carriers of opposite charge thermally occupying states of the same symmetry:

$$k_{\mathrm{rad}}=B^{’}n_{e}^{S}n_{h}^{S}+\frac{1}{3}B^{’}n_{e}^{P}n_{h}^{P}$$ 6

where *n*_*i*_^*j*^ is the occupation of charge carrier *i* in level *j* and *B*′ is chosen such that the calculated total radiative decay rate of the uncharged biexciton matches the experimental value. The exact solution for discrete carrier numbers can be obtained via our microstate model (Supporting Information, Section S8.1). Instead, to produce continuous lines (Figure 4e), we use the well-known Fermi–Dirac distribution function *n*_*i*_^*j*^ = 1/[e^(*E*_*i*_^*j*^ – μ_*i*_)/*k*_B_ *T*^ + 1], which is a decent approximation even for systems with down to 1–4 fermions (Supporting Information, Section S8.2 and ref 46). We isolate the radiative decay rates through the SS and PP transitions:

$$k_{\mathrm{rad}}^{\mathrm{SS}}=k_{\mathrm{rad}}f^{\mathrm{SS}},\mathrm{and},k_{\mathrm{rad}}^{\mathrm{PP}}=k_{\mathrm{rad}}f^{\mathrm{PP}}$$ 7

where *f* ^SS^ and *f* ^PP^ are the fraction of emitted photons in the low-energy and high-energy peak, respectively. These fractions are obtained from a least-squares fit of the PL spectrum using a double Lorentzian model (Figure 1d,e). The experimental *k*_rad_^SS^ and *k*_rad_^PP^ (Figure 4f,g) follow the expected trends

$$k_{\mathrm{rad}}^{\mathrm{SS}}=B^{’}n_{e}^{S}n_{h}^{S},\mathrm{and},k_{\mathrm{rad}}^{\mathrm{PP}}=\frac{1}{3}B^{’}n_{e}^{P}n_{h}^{P}$$ 8

where *n*_*i*_^*j*^ is the Fermi–Dirac occupation of charge carrier *i* in level *j*. The prefactors *B*′ are equal for the two model lines in Figure 4f,g, again demonstrating that the SS and PP transitions have similar oscillator strength (Supporting Information, Section S8.3). A deviation between experiment and model is apparent for *k*_rad_^SS^ at *n*_e_ = 3 and 4, which we ascribe to reduced electron–hole overlap due to electron–electron repulsion.

The scaling of the nonradiative decay rates (Figure 4h) demonstrates that they are due to three-carrier recombination process, most likely Auger recombination. We fit the nonradiative decay rates to

$$k_{\mathrm{Auger}}=C^{-}n_{e}n_{h}\left(n_{e}-1\right)+C^{+}n_{e}n_{h}\left(n_{h}-1\right)$$ 9

where *C* ^–^ and *C* ^+^ are prefactors for the negative- and positive-trion Auger pathway, respectively.^34^ The good match of this expression with the experiment indicates that S and P levels are active in Auger processes at a similar rate. Simpler models used for bulk semiconductors, which disregard the discrete number of charge carriers per BNC, fail to match the experimental data (Figure S11), highlighting the effects of discrete and finite carrier numbers on nonradiative Auger decay. The parameter values are *B*′ = 58.3 μs^–1^, *C* ^–^ = 1.68 μs^–1^, and *C* ^+^ = 3.96 μs^–1^ if the charge densities are expressed in terms of number of charge carriers; or *B*′ = 1.13 × 10^–10^ cm^3^ s^–1^, *C* ^–^ = 6.40 × 10^–30^ cm^6^ s^–1^, and *C* ^+^ = 1.50 × 10^–29^ cm^6^ s^–1^ if converted to units of number of charge carriers per unit of volume, considering the entire BNC core/shell volume.

Our conceptual framework for the energetics of BNCs naturally incorporates elements from existing models: the discrete states from strongly confined NCs with the Fermi–Dirac thermal occupations of a macroscopic semiconductor. Emission from thermally populated levels is likely observable in large nanocrystals of various other materials. The main assumption behind our interpretation and modeling, is the description of excited states in terms of single-carrier excitations separately: the P–P emission arises from an excited electron in the conduction band recombining with an excited hole in the valence band. This description is valid if exciton binding is weak compared to the thermal energy. Exciton binding by Coulomb attraction scales with the reduced exciton mass and inversely with the permittivity of a material. It is similar to or weaker than CdSe for various II–VI, III–V, and VI–IV semiconductors, including CdS,^12^ InP, InAs or PbS. Room-temperature thermally excited emission may be expected for NCs of these materials in the transitional size regime between quantum-confined and bulk. Large halide perovskite NCs have shown no indication of emission from thermally excited states.^47^ This is likely due to a larger exciton binding energy in these materials,^48^ enhanced by polariton formation. The behavior reported in our study is not expected for other materials with more strongly bound excitons. These include any 2-dimensional material with dielectric enhancement of Coulomb attraction, such as colloidal semiconductor nanoplatelets^49^ and monolayer transition metal dichalcogenides;^50^ high-bandgap semiconductors such as oxides or halides;^51^ or materials exhibiting exciton self-trapping, such as CuInS_2_.^52^

A distribution of oscillator strength over an extended spectral range may be an undesired aspect of BNCs—compared to smaller NCs—for application as a laser gain material or a phosphor material. On the other hand, the finite degeneracy of the lowest-energy state, similar to smaller NCs, makes it easier to achieve population inversion compared to bulk materials. New applications of thermally excited emission could include optical cooling^53^ or background-free anti-Stokes microscopy.^54^

The emission efficiencies of multicarrier states in BNCs are spectacularly high, as quantified in our work for isolated states with up to four electrons. Qualitatively, such high emission efficiencies are in line with the volume dependence of undesired Auger recombination.^33,35,45^ Despite the large BNC volumes, statistical-scaling relations still reflect the involvement of discrete charge carriers rather than a continuous charge carrier density. Figure 5 summarizes the emission efficiencies, lifetimes and P emission fractions of the six multicarrier states studied experimentally (Figure 5a,c,e) and the values predicted by our comprehensive model and extrapolated to even higher multicarrier states (Figure 5b,d,f and eqs 8 and 9). The excellent agreement highlights the strength of our model, as it predicts the efficiencies of numerous excited states. The high emission efficiencies and long lifetimes of multicarrier states make BNCs an ideal solution-processable materials class for optoelectronic applications.^11,12^ Auger quenching is limited even for states with as many as four electrons, highlighting the potential for zero-threshold lasing by intentional charging.^26,27^ Our framework provides a predictive and intuitive tool for interpreting the optical behavior of large NCs, as they become increasingly relevant for modern optoelectronic and photonic applications.

**Figure 5:**
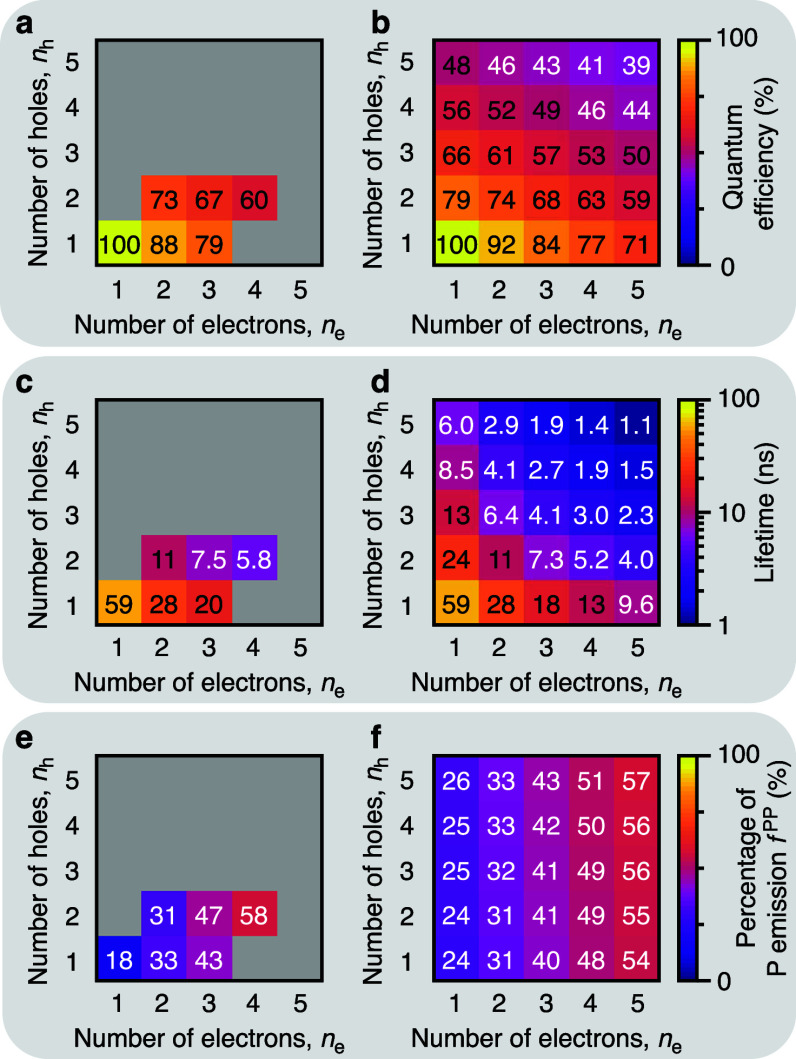
Emission efficiencies and lifetimes of multicarrier states in bulk nanocrystals. (a) Experimental quantum efficiencies of multicarrier states in the BNC studied in Figures 1 and 4, as a function of the number of electrons *n*_e_ and the number of holes *n*_h_. The emission efficiency of the neutral exciton is 100% by assumption, justified by previous photonic experiments on single semiconductor particles.^43^ The ensemble quantum yield is only 20%, likely because of 80% dark BNCs. (b) Same but calculated using the discrete microstate model (Supporting Information, Sections S8.1, S8.3, and S8.4) and using the radiative and Auger recombination rates determined in Figure 4. The model is extrapolated to multicarrier states with up to 5 electrons and 5 holes. (c–d) and (e–f) Same as panels a and b but showing the lifetime and the P emission fraction of multicarrier states, respectively.

## Conclusions

To summarize, we have measured and characterized the energetics and dynamics of six distinct multicarrier states in a single BNC, corresponding to configurations with one or two holes and up to four electrons. We assign the observed bimodal emission spectrum to thermally coupled emission from the 1S–1S and 1P–1P states. This conclusion is supported by temperature-dependent multiparticle spectroscopy, where particle-to-particle size variations yielded a correlation between the peak intensity ratio *R* and the energy difference Δ*E* that follows the expected Boltzmann distribution. Moreover, these measurements revealed that the correlation between the optical bandgap energy *E*_S_ and Δ*E* can be captured by a simple particle-in-a-sphere model. This enabled us to construct a model that predicts both the dynamics and energetics of particles spanning the regime from strongly confined to bulk behavior, in good agreement with our single-particle data.

## Experimental Methods

### Chemicals

Cadmium oxide (CdO, 99.5+%), toluene (C_7_H_8_, 99.9%), methanol (CH_3_OH, 99.8+%) and 2-propanol (C_3_H_8_O, 99.8%) were purchased from Chemlab Analytical. Elemental selenium (Se, 200 mesh) and cadmium acetate dihydrate (Cd­(CH_3_COO)_2_·H_2_O, 98% for analysis) were purchased from Acros Organics. Elemental sulfur (S, ≥ 99.5%) was purchased from Sigma-Aldrich and tri-n-octylphosphine (C_24_H_51_P, min. 97%) was purchased from Strem Chemicals. Squalane (C_30_H_62_, 98%) and oleic acid (C_18_H_34_O_2_, tech. 90%) were purchased from Thermo Fischer Scientific. All chemicals were used as is with no further purifications.

### Slow-Injection Synthesis of CdSe/CdS BNCs

The 10.5/15.5 nm particles were synthesized using the same procedure described in reference 11.

The Cd-oleate (0.50 M) solution was prepared as follows: in a 50 mL three-neck flask, 1.024 g cadmium oxide (8 mmol), 8 mL oleic acid (25 mmol) and 8 mL squalane were added and degassed for an hour at 110 °C. After degassing, the flask was set under inert conditions and heated to 300 °C for around 5–10 min. As soon as the solution turned clear, it was let to naturally cool down to <120 °C and then collected.

The TOP-Se (0.50 M) and TOP-S (0.50 M) solutions were prepared in separate 20 mL vials inside a N_2_-filled glovebox. Ten mL tri-n-octylphosphine (22.4 mmol) were added to 0.395 g selenium (5 mmol) and 0.160 g sulfur (5 mmol), respectively. The vials were left to stir at 90 °C for around 30 min until the solutions turned clear.

For the slow - injection synthesis, 8 mL squalane were added in a 50-ml three-neck flask and degassed at 110 °C for an hour. After degassing, the flask was filled with N_2_ and heated to 340 °C. In the meantime, an equimolar mixture of preheated Cd-oleate and TOP-Se was prepared in a glovebox. The mixture was inserted in a 5-ml syringe and slowly injected into the flask using a 2 mL/h rate. The total injection time is modified as needed to produce the desired core size. For the 10.5 nm CdSe core, a total of 0.5 mL Cd-oleate and 0.5 mL TOP-Se were mixed and injected over the course of 60 min. After the injection, the flask was held at 340 °C for around 10 min to allow for full precursor consumption.

To form the CdS shell with a 2.5 nm thickness, a 5-ml syringe filled with an equimolar solution of 0.75 mL Cd-oleate and 0.75 mL TOP-S was similarly prepared. In a one-pot process, the second solution was injected at 340 °C with the same rate into the reaction flask over the course of 90 min. After the injection, the flask was held for an additional 30 min at 340 °C to allow for full precursor consumption.

For the purification, the cooled-down crude medium was split into four centrifugation tubes containing a 2:1 ratio mix of 2-propanol and methanol (around 15 mL in each tube). The tubes were then centrifuged at 5000 rpm for 10 min to precipitate the particles. This process was repeated for a total of two cycles. The particles were then redispersed in 3 mL toluene for further use.

An acetate ligand post-treatment was finally implemented to help increase the particle luminescence. For this, 100 μL of oleic acid and 0.0268 g of grinded cadmium acetate dihydrate were added in a 20 mL vial along with the full purified BNC batch. The partially open vial was then sonicated at 80 °C for 90 min. After sonication, the vial was centrifuged at 5000 rpm for 15 min to remove any unreacted salts and coagulated particles. The sur natant was collected and filtered in a new vial, where 6 mL 2-propanol were added to start a mild purification step. The particles were crashed by centrifuging at 5000 rpm for 3 min and finally redispersed in 3 mL toluene.

### Characterization

The core/shell dimensions of the BNCs were then verified through bright-field TEM imaging. The images were obtained on a JEOL JEM-1011 microscope equipped with a thermionic gun operating at 100 kV accelerating voltage. Image processing was done using ImageJ software. Particle sizing was performed using a Python script based on a binary thresholding model that distinguishes particles from the background by pixel intensity. After image filtering and thresholding, the particles were identified, size-filtered, and scaled to nm. Assuming spherical geometry, the average diameters were calculated, and size distributions were generated from over 300 particles (Figure S1).

### Cascade Spectroscopy

Single bulk nanocrystals (BNC) measurements were conducted using a custom-built microscope setup based on a Nikon–Ti-U inverted microscope.

The sample was prepared by spin-coating a highly diluted solution (1:20,000 dilution compared to the stock) onto a glass coverslip. This coverslip was then mounted onto a microscope slide under a nitrogen atmosphere using an airtight adhesive spacer, effectively excluding oxygen and stabilizing excess electrons in the BNC.

BNC excitation was achieved using a pulsed 405 nm laser (Picoquant D-C 405, controlled by Picoquant PDL 800-D laser driver operating at 1 MHz), which was guided to the sample by a dichroic mirror (425 nm, Thorlabs DMLP425R). For single-particle measurements, the light was focused through a high-numerical-aperture oil-immersion objective (Nikon CFI Plan Apochromat Lambda 100×, NA 1.45). Emission from the sample was collected through the same objective and spectrally dispersed using a transmission grating (Thorlabs, 300 lines/mm). With an achromatic aspherical lens (Edmund, *f* = 50 mm) the Fourier plane of the transmission grating was then imaged onto a one-dimensional single-photon avalanche photodiode (SPAD) array consisting of 320 pixels (SPAD Lambda, Pi Imaging), achieving a spectral resolution of 1.84 nm/pixel. To eliminate residual laser light, a long-pass filter (430 nm, Bright line) was placed in the detection path. Based on the count rate relative to a calibrated setup^28^ with two single-pixel SPADs, we determine a detection efficiency of approximately 4%. The excitation rate μ for the experiments in the main text is ∼0.2 excitations per laser pulse.

### Temperature-Dependent Multiparticle Spectroscopy

For the temperature-dependent multiparticle experiments, the excitation path was modified by inserting an additional 20 cm-focal-length lens to focus the laser onto the back focal plane of the objective, enabling widefield illumination. The sample preparation followed the same protocol as for the single-particle measurements, with the exception that the BNCs were spin-coated onto a Vaheat temperature-controlled substrate (Interherence, maximum temperature 100 °C) instead of a glass coverslip. To minimize multiexciton generation and suppress charging effects, the measurements were performed under low-intensity continuous-wave (CW) excitation at 405 nm and under ambient conditions. Prior to data acquisition at higher temperatures, the sample was heated (to 60 °C) and allowed to thermally stabilize for 5 min. The emitted photoluminescence was dispersed by a spectrograph and recorded using an electron-multiplying CCD camera. Time-averaged PL spectra were acquired using the following settings: EM gain 300, slit width 50 μm, 100 accumulations, and 0.1 s exposure time per frame.

## Supplementary Material



## Notes

Details on synthesis, sample preparation, single-particle and temperature-dependent multiparticle spectroscopy methods, data analysis, derivations of the microstate and statistical models, and extended data on a second BNC (PDF)
