# Supplementary material for: Vanishing Quantum Confinement Enables Bright and Thermally Excited Multicarrier Emission from Semiconductor Nanocrystals
Source: ACS Nano. 2026 Jan 20;20(4):3845–54. doi: 10.1021/acsnano.5c19908 (PMC13450707; doi:10.1021/acsnano.5c19908)
Supplement: Supplementary file 1 [file nn5c19908_si_001.pdf]

Supporting Information for:

Vanishing quantum confinement enables bright and thermally excited multi-carrier emission from semiconductor nanocrystals

Tjom Arens[1,2], Sander J.W. Vonk[3], A. Willem Vlasblom[1], Margarita Samoli[4], Daniel Vanmaekelbergh[2], Pieter Geiregat[4,5], Zeger Hens[4,5] and Freddy T. Rabouw[1,2,*]

[1]*Soft Condensed Matter & Biophysics, Debye Institute for Nanomaterials Science, Utrecht University, Princetonplein 1, 3584CC Utrecht, The Netherlands*

[2]*Condensed Matter & Interfaces, Debye Institute for Nanomaterials Science, Utrecht University, Princetonplein 1, 3584CC Utrecht, The Netherlands*

[3]*Optical Materials Engineering Laboratory, Department of Mechanical and Process Engineering, ETH Zurich, 8092 Zurich, Switzerland*

[4]*Physics and Chemistry of Nanostructures, Department of Chemistry, Gent University, Krijgslaan 281, 9000 Gent, Belgium*

[5]*NOLIMITS, Core Facility for Non-Linear Microscopy and Spectroscopy, Gent University, Krijgslaan 281, 9000 Gent, Belgium*

*Corresponding author: f.t.rabouw@uu.nl

S1 Synthesis and characterisation of bulk nanocrystals

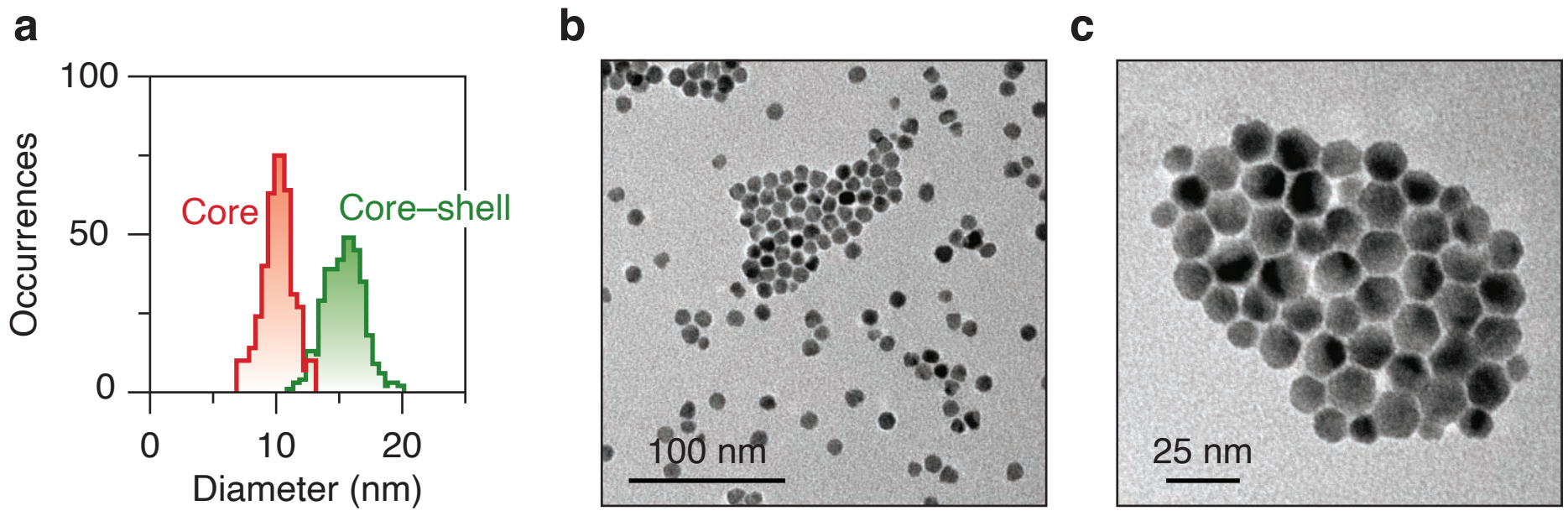

Figure S1 | Particle size analysis of CdSe/CdS BNCs. (a) Size distributions of CdSe cores (red) and CdSe/CdS core–shell BNCs (green) obtained from TEM. Average diameters: 10.1 ± 1.2 nm (core) and 15.4 ± 1.5 nm (core–shell). **(b–c)** Representative TEM images of CdSe/CdS BNCs.

S2 Effect of oxygen on single-BNC charging

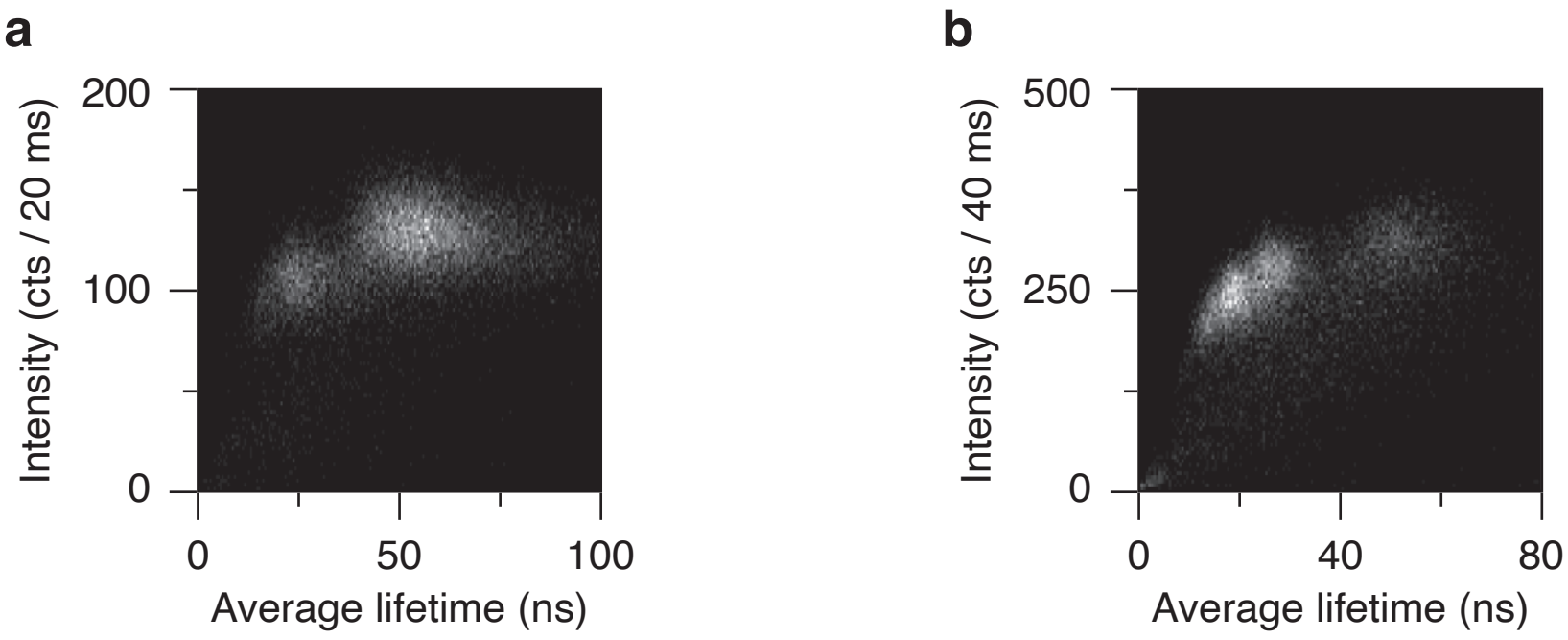

Figure S2 | Single-BNC measurement acquired in ambient and nitrogen atmospheres. Fluorescence-lifetime–intensity distribution for a single-BNC measurement recorded under **(a)** ambient conditions and **(b)** under a nitrogen atmosphere. Charging events are more pronounced in the nitrogen environment, consistent with the role of oxygen as an efficient electron scavenger that suppresses long-lived charged states.

S3 SPAD array wavelength calibration

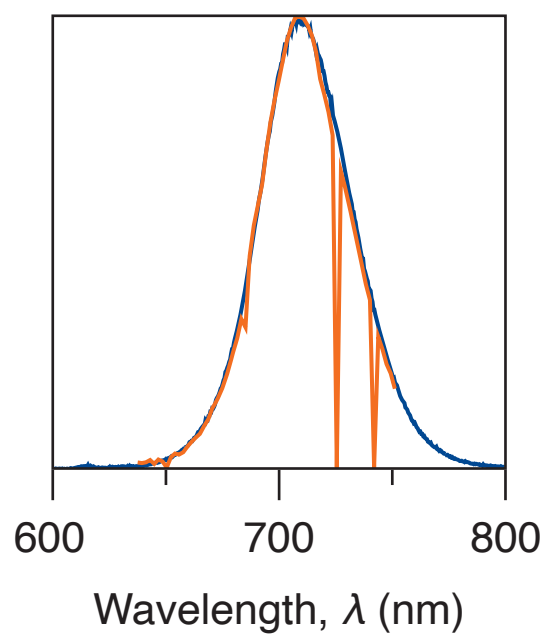

Figure S3 | Wavelength calibration of the time-correlated spectrometer. To calibrate the wavelength recorded on each pixel of our time-correlated spectrometer, we dropcasted a 10× diluted BNC stock solution on a glass coverslip and measured the emission of a thick layer on a glass substrate. A flipper mirror was used to alternate the emission path between an electron-multiplying CCD (Andor iXon Ultra 888) with a calibrated spectrograph (Andor Kymera 193i) and the SPAD array. By overlaying the photoluminescence (PL) spectra from both detectors, we assigned wavelengths to individual SPAD pixels. Two hot pixels with extreme dark-count rates were identified and rejected from all further analysis in the single-BNC experiments. This procedure yielded a spectral resolution of 1.84 nm per pixel for the SPAD setup.

S4 Blind-time analysis

Conventionally, the observation of anti-bunching, defined as $g^{(2)}(0) < 0.5$ in the normalised correlation function $g^{(2)}(\tau)$, is regarded proof of a single emitter in the detection volume of a spectroscopy setup. However, the high multiexciton emission efficiencies of BNCs cause reduced anti-bunching, such that $g^{(2)}(0) > 0.5$ even for a single BNC. To assess whether the emission originates from a single BNC or a cluster of emitters, we therefore employed a blind-time analysis based on $g^{(2)}$. This method uses the difference in the characteristic lifetimes of the two photons in a cascade event, a biexciton photon followed by an exciton photon. Since the first photon of a cascade (BX) has a relatively short lifetime compared to a photon emitted from an exciton state (X), applying a short blind period (Δ) after a laser pulse rejects coincidence counts originating from a cascade more strongly than coincidences from two independently emitted exciton photons.[3]

If we study a single BNC with high biexciton emission efficiency, the coincidence counts in the zero-delay peak are due to cascade emissions. Because the exciton photon always follows the biexciton photon the number of photon pairs in the zero-delay peak is proportional to the number of biexcitons that are emitted outside the blind period:

$$g^{(2)}(0, \Delta) \propto \exp\left(-\frac{\Delta}{\tau_{\mathrm{BX}}}\right) \tag{S1}$$

In contrast, the side peaks (at ±1 pulse repetition period) reflect uncorrelated exciton photons from subsequent excitation cycles—in the low excitation regime. The number of photon pairs in the side peak as a function of the blind window scales as:

$$g^{(2)}(\pm T, \Delta) \propto \left[\exp\left(-\frac{\Delta}{\tau_{\mathrm{X}}}\right)\right]^2 = \exp\left(-\frac{2\Delta}{\tau_{\mathrm{X}}}\right) \tag{S2}$$

By comparing the decay behaviour of correlated photon pairs as a function of Δ, one can infer whether the observed emission is consistent with a single BNC or a collection of emitters (Figure S4).

Fitting the peak amplitude as a function of blind time for the zero-delay and side peak yields a biexciton lifetime $\tau_{\mathrm{BX}} = 10.8$ ns and an exciton lifetime $\tau_{\mathrm{X}} = 47.2$ ns, respectively. These values are similar to the values that we obtained from the time-averaged decay curves ($\tau_{\mathrm{BX}} = 11.2$ ns and $\tau_{\mathrm{X}} = 58.9$ ns), indicating that the collected emission originated from a single BNC.

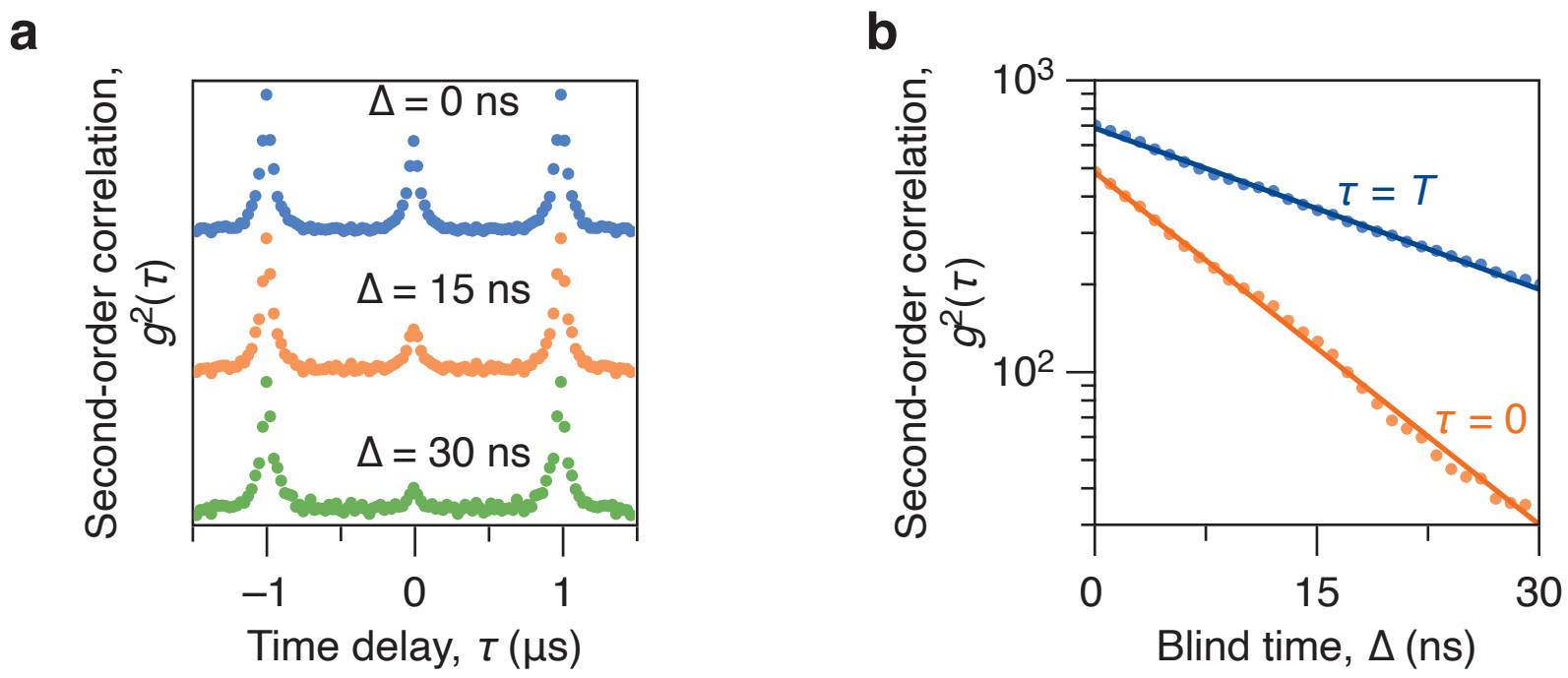

Figure S4 | Determining the number of emitters via blind-time analysis. (a) Second-order correlation function $g^{(2)}(\tau)$ of the neutral exciton, calculated while omitting all photons arriving within $\Delta = 0$ ns (blue), 15 ns (orange), and 30 ns (green) after each laser pulse. Increasing the blind time rejects photon pairs due to fast biexciton emission from the biexciton cascade more strongly compared to pairs from consecutive exciton photons. As a result, the zero-delay peak is expected to decrease more rapidly than the side peaks. **(b)** Amplitude of the zero-delay peak (orange) and the side peak (blue) as a function of blind time. The zero-delay peak decays more rapidly than the side peak, consistent with the shorter lifetime of the biexciton. This behaviour indicates that the emission originates from a single emitter rather than a cluster of emitters.

S5 State selection and excited-state characterisation

To isolate six multi-carrier states from the continuous photon stream emitted by a single BNC, we identify the number of excess charges in the BNC for each 40-ms time bin of the experiment by lifetime thresholding and subsequently distinguish between exciton and biexciton photons through selection of cascaded emission events.

S5.1 Charged-state selection

To obtain the intensity and lifetime for each 40-ms time bin over the course of our experiment, we applied maximum-likelihood estimation (MLE) to fit the corresponding decay curves, accounting for the Poissonian statistics of photon counting. This procedure yielded both the average fluorescence lifetime and emission intensity per bin, which were subsequently used to construct the fluorescence-lifetime–intensity distribution (FLID; Figure 1b in the main text).

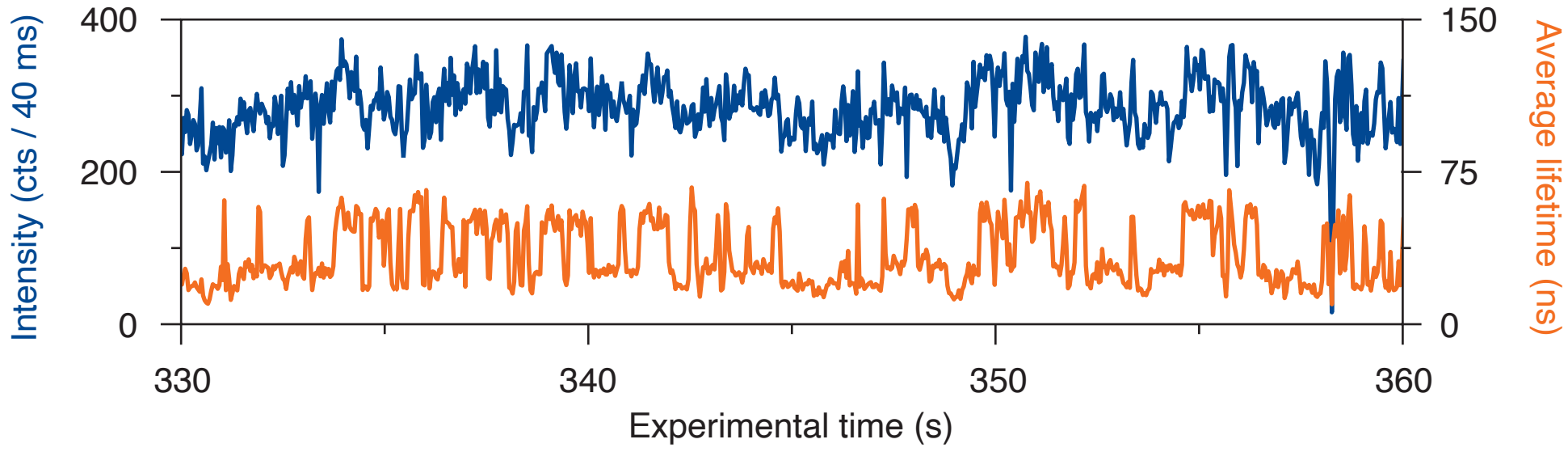

Figure S5 | Blinking trace showing fluctuations in intensity and lifetime due to spontaneous charging. A segment of the experiment from the main text is shown. The intensity trace exhibits multiple emission levels without distinct boundaries, whereas the corresponding average lifetime trace reveals clear transitions between different charge states.

Periods of BNC charging are characterised by a lower count rate and shorter excited-state lifetime, because of increased non-radiative and radiative recombination pathways (Figure S5). Previous studies on smaller QDs often made the distinction easy because the efficiency of the charged (OFF) state was relatively low compared to the neutral (ON) state. Our BNCs, however, have charged-state emission efficiencies above 70%. This makes separation of charged and neutral periods based on intensity almost impossible. We therefore separate the states based on their average lifetime, instead.

A histogram of the average lifetimes across 40-ms bins (Figure S6) reveals three peaks centred at 51 ns, 26 ns, and 18 ns, corresponding to neutral, singly charged, and doubly charged states. A weaker shoulder appears around 12 ns, which most likely corresponds to a state with three additional electrons, but its low population and partial overlap with other distributions make it unreliable to separate quantitatively. For this reason, we exclude it from our analysis.

Bins outside these dominant lifetime intervals arise mainly from flickering, where the BNC switches between two charge states within a single time bin, and from spectral diffusion. To minimize such contributions, narrow lifetime windows were manually selected around the main peaks (±6 ns for the neutral state, ±3 ns for the singly and doubly charged states; Figure S6), ensuring analysis of bins with well-defined photophysical character while maintaining sufficient photon statistics.

In our experiments the laser fluence remains constant and we assume the absorption cross section to remain constant. We can therefore determine the emission efficiency of a charged state from the average counts (per 40 ms) compared to the neutral state, which we assume to have unity emission efficiency.[4]

With each photon assigned to specific charged states, we can reconstruct the photoluminescence (PL) spectra and time-averaged PL decay curves. We normalise each decay curve to the time spent (number of 40-ms time bins) in a certain state and each spectrum to its emission maximum.

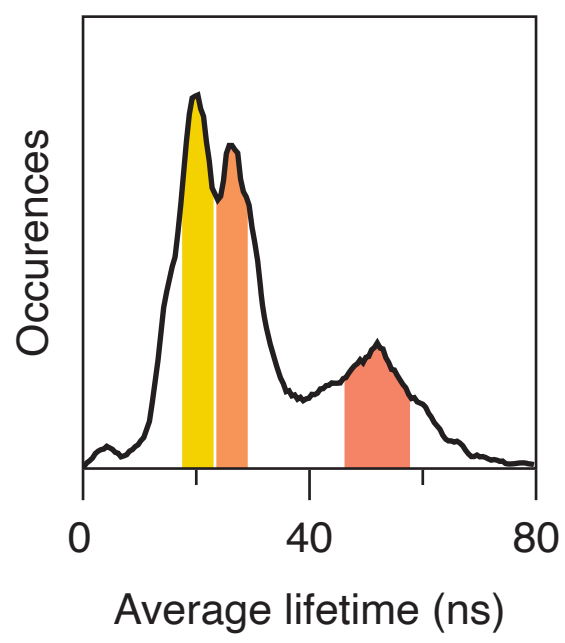

Figure S6 | Lifetime histogram revealing multiple emitting states. Histogram of the average lifetimes of photons within 40-ms time bins. Four distinct peaks are observed, corresponding to an off state (4 ns), neutral exciton state (51 ns), singly charged state (26 ns), and doubly charged state (18 ns). Note that the average-lifetime values are slightly shorter than the (un)charged exciton lifetimes determined in Figure 4 of the main text, because the calculation of average lifetime includes some (un)charged biexciton photons.

S5.2 Isolation of biexciton cascade events

The selection of cascade events from the photon stream can lead to false cascade detections on our SPAD array. First, despite a low dark count rate per pixel ($k_D < 100$ s^{-1}), the spectral dispersion over ~40 pixels increases the likelihood of cascade events containing at least one dark count. Second, pixels can re-emit photons after absorption, causing crosstalk detections on neighbouring pixels.[5,6] To reduce these sources of false positives, we apply time gating to the first and second photon of each cascade event based on the lifetime of the exciton and biexciton of each state. The first photon of a cascade event should be detected within approximately 3 times the (un)charged biexciton lifetime. For the BNC analysed in the main text, these are 33, 23 or 18 ns for the neutral, singly charged or doubly charged biexciton, respectively. Similarly, the second photon of the cascade should be detected within 3 times the (un)charged exciton lifetime, i.e. within 170, 84 or 60 ns after detection of the first photon. These upper limits of the time gates help us reject dark counts occurring over the full laser repetition period. We put a lower limit of 1 ns on the delay time between first and second photon to reject crosstalk events. After this time-gating procedure on the photon cascades, we can reconstruct the (un)charged-biexciton PL decay curves and emission spectra by analysing all first photons of the cascade events.

We scale the (un)charged-biexciton PL decay curves in the main text, such that the amplitude is proportional to the radiative decay rate. Three correction factors were applied:

1. Residence time (number of time bins) in the given charge state,
2. Emission efficiency for the second photon in the cascade (i.e., the (un)charged exciton emission efficiency), and
3. Loss of second-photon events due to the time-gating procedure on cascade events.

For (3), we estimated the fraction f of second photons that were accepted by time gating with lower limit $t_1 = 1$ ns and and upper limit t_2 (170, 84, or 60 ns for the BNC in the main text):

$$f = \int_{t_1}^{t_2} \frac{1}{\tau} \mathrm{e}^{-t/\tau}\, \mathrm{d}t = \mathrm{e}^{-t_1/\tau} - \mathrm{e}^{-t_2/\tau}, \tag{S3}$$

where τ is the fitted lifetime of the (un)charged exciton state. Using these corrector factors, the (un)charged-biexciton PL decay curves show the expected trend of increasing radiative rate with increasing number of uncompensated charges in the BNC.

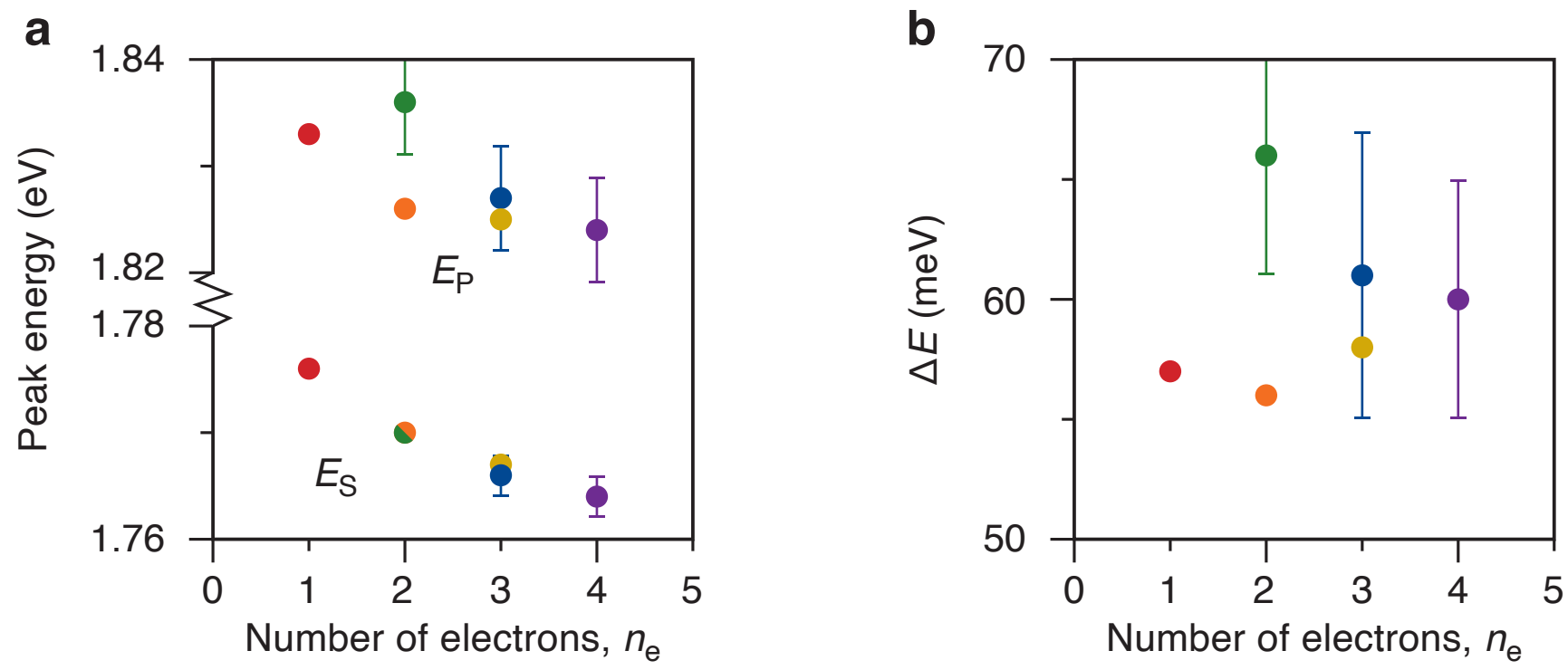

Figure S7 | Peak energies and S–P energy gap of different multi-carrier states. (a) Fitted emission peak positions E_S and E_P on the multi-carrier spectra isolated in Figure 1 of the main text, plotted against the number of electrons in a state. The colors are the same as used in the main text. Error bars indicate $\pm 2\sigma$, with σ the standard error on the fit. If errors are not shown, they are smaller than the size of the data point. **(b)** The S–P energy gap, as a function of the number of electrons in a state.

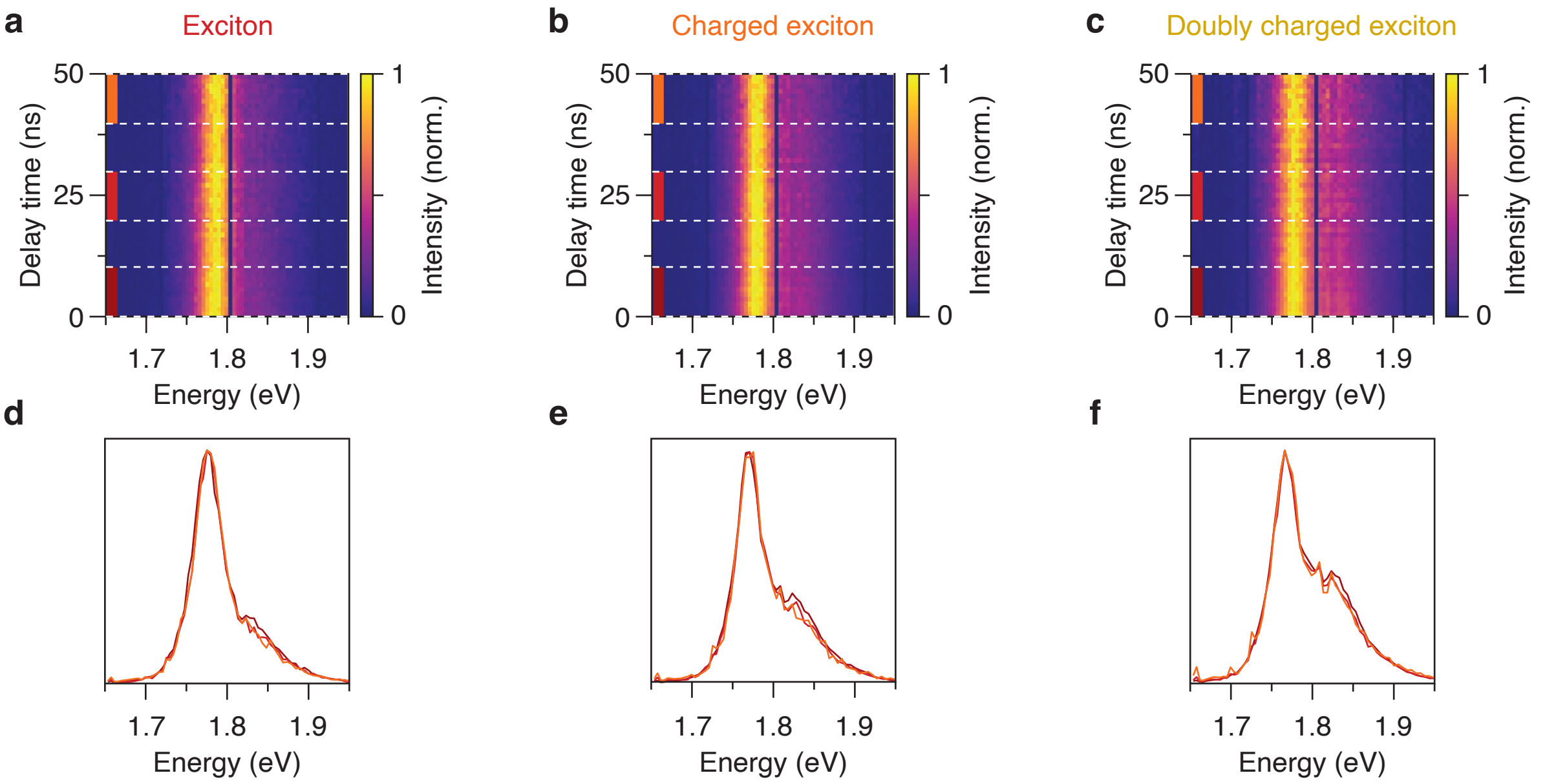

Figure S8 | Time-resolved emission maps of the different charge states. Normalised time-resolved emission maps showing the spectral evolution of **(a)** the neutral exciton, **(b)** the singly charged exciton, and **(c)** the doubly charged exciton, as a function of delay time. Spectra integrated over delay ranges of 0–10, 20–30 and 40–50 ns for **(d)** the neutral exciton, **(e)** the singly charged exciton, and **(f)** the doubly charged exciton. Over the first 10 ns, a slight decrease in the higher-energy P-level contribution is observed, which stabilises at longer decays. This behaviour is attributed to the presence of the (un)charged biexciton, which has a shorter lifetime than the (un)charged exciton and makes a contribution only at short times. At later delay times, when only exciton emission remains, the spectral shape remains unchanged. These results indicate that the decay from the two emitting levels is thermally coupled.

S5.3 Biexciton lifetimes

Because of the limited number of detected photons in the cascaded PL decay curves (~1000 counts), we again employ a maximum-likelihood estimation (MLE) fitting procedure to obtain the biexciton lifetimes τ_{BX}. MLE provides a more accurate estimation than least-squares fitting in the low-count regime, as it accounts for the Poissonian nature of photon-counting statistics.[7,8]

We assume single-exponential decay:

$$I(t) = \frac{1}{\tau} \exp\left(-\frac{t}{\tau}\right) \tag{S4}$$

To account for the instrument response function (IRF), we convolve this decay with a Gaussian IRF with standard deviation σ and offset t_0, yielding the analytical form:

$$I_{\text{conv}}(t;\tau) = \frac{1}{N} \exp\left(-\frac{t-t_0}{\tau}\right) \operatorname{erfc}\left(\frac{\sigma^2 - (t-t_0)\tau}{\sqrt{2}\,\sigma\,\tau}\right) \tag{S5}$$

where erfc(t) is the complementary error function, and N is a normalisation factor:

$$N = \tau\left[\exp\left(-\frac{\sigma^2}{2\tau^2}\right) + \operatorname{erfc}\left(\frac{\sigma}{\sqrt{2}\,\tau}\right)\right] \tag{S6}$$

Under Poisson statistics the negative log-likelihood function is given by:

$$\mathcal{L} = -\sum_i (y_i \ln I_i - I_i) \tag{S7}$$

where $I_i = I_{\text{conv}}(t_i;\tau)$ is the expected number of photon counts at delay time t_i according to the model and y_i is the measured number of photons. We minimise $\mathcal{L}$ with respect to τ and scale the normalised model to match the total photon count in our histogram.

S5.4 Exciton lifetimes

To disentangle the contributions of exciton and biexciton emission in the total time-averaged decay curves, we performed biexponential fits. The total decay is modeled as a sum of two single-exponential components: a fast decay attributed to biexciton recombination and a slower decay corresponding to exciton emission. We use the independently determined biexciton lifetimes for each charge state as fixed input parameters for the fast component in the biexponential fits. This constraint ensures that the extracted amplitude and lifetime of the slower component more accurately reflects the properties of the exciton. To minimise distortion from delayed emission due to reversible charge carrier trapping[8], we restricted the fit to the linear portion of the decay curve on a logarithmic scale. The fitting window was selected manually for each charge state through visual inspection. Specifically, we used the first 125 ns for the neutral state, 75 ns for the singly charged state, and 60 ns for the doubly charged state.

S5.5 Emission efficiencies

In our experiments, the laser fluence is kept constant. As excitation occurs at high energy (405 nm), we assume that the absorption cross section is independent of charge state. The emission efficiency of a charged exciton state is therefore determined from its photon count rate (centre of a Gaussian fit to the count-rate histogram) relative to that of the neutral exciton, which has been determined to have unity efficiency with previous photonic experiments on single semiconductor particles.[4]

To evaluate the emission efficiency of the (un)charged biexciton cascade, we analyse the second-order correlation function $g^{(2)}$. We extract the biexciton-to-exciton emission ratio by fitting the correlation histogram with three double-sided exponentials with equal lifetimes, corresponding to the exciton lifetime, but allowing for independent amplitudes for the central (zero-delay) and side peaks. The data point at a delay time of to $\tau = 0$ is excluded from the fit, as our crosstalk correction introduces an artificial suppression of photon-pair detections at $\tau = 0$. We multiply the amplitude ratio from the fit with the emission efficiency of the (un)charged exciton state to obtain the emission efficiency of the (un)charged biexciton state.

S6 Analysis of multi-particle spectroscopy

To extract spectral features from individual BNCs, we implemented a multistep correction and fitting procedure. First, we corrected for slight curvature in the spectrometer slit by centering each row of the EMCCD image. This alignment was based on a pixel shift derived from a reference measurement of the laser reflection at 405 nm spectrally dispersed on the camera. Following this, a flat background was subtracted from each row on the EMCCD image using the average signal in the leftmost 20 pixels, which lay outside the BNCs emission range.

To compare spectra recorded at different temperatures, we accounted for potential vertical shifts. This was achieved by shifting images with respect to each other to maximise the correlation between the summed intensity profiles of each image.

We then identified particle positions based on the high-temperature measurement, using both a minimum and maximum intensity threshold to select bright, well-isolated particles. This approach thus excluded BNCs that had photobleached or gone dark at elevated temperature.

To accurately describe the PL spectra of single BNCs, we fitted the data using a sum of three Gaussian functions with equal width (σ). While most BNCs exhibited two main peaks corresponding to the S and P exciton state, a subset displayed an additional, red-shifted emission peak. We attribute this third peak to emission from a trap state and excluded it from our analysis. For each selected BNC, we initially performed an unconstrained fit. From this set of preliminary fits, we computed the average σ across all particles and used this as a fixed parameter in a second, constrained fit for each spectrum. This final fit yielded robust estimates of the energies of the two dominant transitions E_S and E_P, their energy separation ΔE_{P-S}, and the intensity ratio $R = I_P/I_S$. To prevent bias due to the quenching of a subset of BNCs at higher temperatures, we consider only BNCs emissive at both temperatures for the analysis of Figure 2 in the main text.

S7 Single-particle quantum confinement model for exciton states

S7.1 S–P confinement energy

In a spherical quantum dot, the confinement energy spacing can be approximated using the particle-in-a-box model. The S–P spacing scales inversely with the square of the confinement radius and inversely with the effective mass of the carrier. For electrons and holes confined over different volumes, the total S–P spacing in the emission spectrum is the sum of the electron and hole contributions:

$$\Delta E = \Delta E_{\mathrm{e}} + \Delta E_{\mathrm{h}} \propto \frac{1}{m_{\mathrm{e}} R_{\mathrm{e}}^2} + \frac{1}{m_{\mathrm{h}} R_{\mathrm{h}}^2} \tag{S8}$$

Here, $m_e = 0.1m_0$ and $m_h = 1.0m_0$ are the effective masses of the electron and hole, respectively. The confinement radii for a typical BNC from our batch are used $R_e = 7.7$ nm for the electron and $R_h = 5.05$ nm for the hole. By inserting these values, we obtain the separate contributions to the S–P level spacing for electrons and holes. These values are used directly in the modelling presented in later Supplementary Notes, where ΔE_{e} and ΔE_{h} enter the discrete and continuous Fermi–Dirac descriptions of multi-carrier state filling.

S7.2 Degeneracy of bright exciton states

Including spin and valence-band multiplicity, there are 2×4 = 8 SS exciton configurations and 6×12 = 72 PP exciton configurations. However, only electron–hole pairs with matching orbital symmetry and orientation are optically bright (e.g., a P_x electron recombining with a P_x hole). This yields a bright PP fraction of 1/3, corresponding to an effective degeneracy ratio of

$$\frac{g_{\mathrm{P}}}{g_{\mathrm{S}}} = 3. \tag{S9}$$

We need this degeneracy ratio for our Boltzmann model (equation (1), main text), which describes the intensity ratio R between bright SS and PP excitons as a function of $\Delta E_{\mathrm{P-S}}$.

S7.3 Relation between the energy-level spacing and the optical bandgap

To describe the experimentally observed correlation between $\Delta E_{\mathrm{P-S}}$ and E_{S} (Figure 2d, main text) we use the particle-in-a-box model for quantum confinement. In this model, the energy of a confined carrier is given by:

$$E = \frac{\hbar^2 \chi_{n,l}^2}{2m^* R^2} \tag{S10}$$

where $\chi_{n,l}$ is the n-th zero of the spherical Bessel function of order l, m^* is the effective mass of the carrier and R is the radius of the confinement. We express $\Delta E_{\mathrm{P-S}}$ in terms of E_{S}, using:

$$\Delta E_{\mathrm{P-S}} = E_{\mathrm{P}} - E_{\mathrm{S}} = (\chi_{11}^2 - \chi_{10}^2)\, \frac{\hbar^2}{2} \left(\frac{1}{m_{\mathrm{e}} R_{\mathrm{e}}^2} + \frac{1}{m_{\mathrm{h}} R_{\mathrm{h}}^2} \right) \tag{S11}$$

and

$$E_{\mathrm{S}} - E_{\mathrm{g}} = \chi_{10}^2\, \frac{\hbar^2}{2} \left(\frac{1}{m_{\mathrm{e}} R_{\mathrm{e}}^2} + \frac{1}{m_{\mathrm{h}} R_{\mathrm{h}}^2} \right) \tag{S12}$$

Taking the ratio of the two expression yields equation (2) of the main text, which is a simple relation between $\Delta E_{\mathrm{P-S}}$ and E_{S}. While the absolute energies E_{S} and E_{P} depend on the asymmetric confinement of the electrons and holes in our BNCs, their difference $\Delta E_{\mathrm{P-S}}$ scales linearly with $E_{\mathrm{S}} - E_{\mathrm{g}}$.

S7.4 Size-dependent emission spectra

We model the expected PL spectra of the neutral exciton in particles with varying degrees of quantum confinement by considering thermal population of excited states using Boltzmann statistics, while obtaining the energy-level spacing based on the effective-mass model. The energy levels are calculated from the solutions to the Schrödinger equation in a spherical hard-wall potential. We use the first 40 zeros of the spherical Bessel functions j_l, with angular momentum quantum number $l \in [0,70]$ and radial quantum number $n \in [1,40]$. Each level's degeneracy is given by $2l+1$, reflecting the number of magnetic sublevels for a given l.

The energy of the lowest transition is calculated using an effective-mass model, assuming the electron is confined over the whole particle ($2R_e$ = 15.5 nm), and the hole is confined to the core ($2R_h$ = 10.5 nm). The electron and hole effective masses are set to $m_e = 0.1m_0$ and $m_h = 1.0m_0$, where m_0 is the free electron mass. This yields the energy for the lowest allowed optical transition in a particle with the average geometry from the synthesis batch:

$$E_{\mathrm{S}} = E_{\mathrm{g}} + \frac{\hbar^2 \pi^2}{2 m_{\mathrm{e}} R_{\mathrm{e}}^2} + \frac{\hbar^2 \pi^2}{2 m_{\mathrm{h}} R_{\mathrm{h}}^2} \tag{S13}$$

The energy difference between the SS and PP exciton for the particle of the average geometry is

$$\Delta E = \left(\frac{\hbar^2}{2 m_{\mathrm{e}} R_{\mathrm{e}}^2} + \frac{\hbar^2}{2 m_{\mathrm{h}} R_{\mathrm{h}}^2} \right) (\chi_{11}^2 - \pi^2) \tag{S14}$$

In Figure 3 of the main text, we model particles with the average core and total size (see above), as well as particles with different sizes. Specifically, we consider particles with confinement energies scaled by factors 0.1, 0.6, 1, 2, 3, compared to the average particle. This represents particles ranging from bulk-like to strongly confined. For each case, we compute the Boltzmann-weighted populations of all 2840 levels and convolve them with Gaussian emission lines (σ = 25.7 meV) centred at each transition energy to simulate the PL spectrum.

We compare the emission spectrum of the smallest simulated particle (topmost in Figure 3d of the main text) with that obtained from a strongly confined model in which thermal excitation to higher energy levels is suppressed and thus all emission originates from the lowest-energy transition. The close agreement between the two spectra confirms that this particle is in the strong-quantum-confinement regime.

In contrast, the spectrum of the largest particle (bottommost in Figure 3d of the main text) is compared with that predicted by a bulk model based on the joint density of states. Specifically, we solve for the electron chemical potential μ_e from the equation for Fermi–Dirac occupation, such that the total number of electrons in delocalization volume $V_e = \frac{4}{3}\pi R_e^3$ (with R_e the total BNC radius) equals $n_e = 1$:

$$\frac{n_e}{V_e} = \int_0^\infty \frac{\sqrt{2m_e^3}}{\hbar^3\pi^2}\sqrt{E}\,\frac{1}{e^{(E-\mu_e)/k_BT}}\,dE \tag{S15}$$

Similarly, the hole chemical potential μ_h is found for a delocalisation volume with core radius R_h and $n_h = 1$ hole. Next, the predicted emission rate as a function of photon energy E is

$$I(E) = A\sqrt{E} \times \frac{1}{1 + \exp\left[\left(\frac{m_h}{m_h + m_e}E - \mu_e\right)/k_BT\right]} \times \frac{1}{1 + \exp\left[\left(\frac{m_e}{m_h + m_e}E - \mu_h\right)/k_BT\right]} \tag{S16}$$

Here, A is a prefactor that we scale to match the bulk and the quantum-confined thermal occupation models. The second factor on the righthand side describes the electron occupation at a vertical transition in the dispersion diagram at energy E and the third factor the hole occupation at E. The predicted bulk emission spectrum, equation (S16), is also broadened by 25.7 meV.

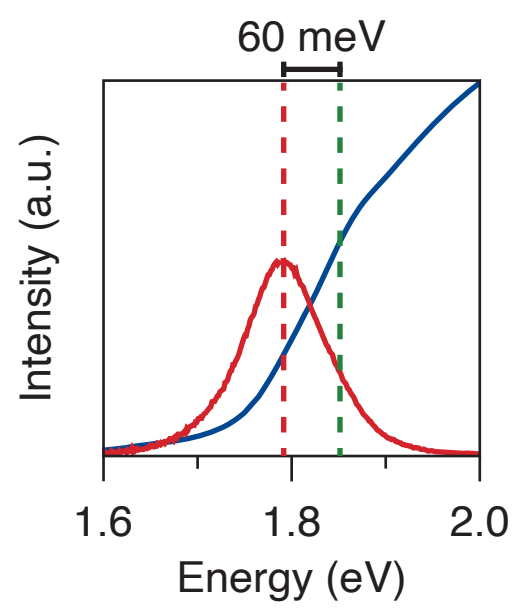

Figure S9 | Quantifying the Stokes shift in bulk nanocrystals. To qualitatively assess the deviation between our measured bandgap—obtained via temperature-dependent multiparticle spectroscopy—and literature values, we plot the ensemble photoluminescence (red) and absorption spectrum (blue). The Stokes shift is approximately 60 meV, consistent with the value of 1.69 eV for the bulk bandgap (Figure 2d of the main text) extrapolated from the emission peak energies.

S8 Fermi–Dirac occupation of electron and hole levels in multi-carrier states

The famous Fermi–Dirac distribution function

$$f(E) = \frac{1}{e^{(E-\mu)/k_B T} + 1} \tag{S17}$$

approximates fermion statistics in the limit of a large number of fermions, valid if the chemical potential μ changes negligibly upon addition of one more fermion.[9] The multi-carrier states studied in the main text, with at most 4 electrons and 2 holes, are not exactly in this limit. Nevertheless, we use this approximation in Figure 4 of the main text and Supplementary Figure 8a–b, where we need a distribution function for a continuous total number of electrons n_e and holes n_h. For the modeling in Figure 3 and 5, in contrast, we use a more exact description in terms of possible microstates of multi-carrier states with integer n_e and n_h. The fermionic nature of electrons and holes is included by restricting the number of particles in S and P levels.

In this section, we will describe the discrete Fermi–Dirac occupation model and compare the predictions to those of Supplementary equation (S17) as well as to an even simpler Boltzmann model for electron and hole occupations.

S8.1 Discrete microstate model

For a specific multi-carrier state with fixed total number of n_e indistinguishable electrons and n_h indistinguishable holes, we consider the distributions of electrons and holes over single-carrier levels separately.

The possible microstates for the electrons form a canonical ensemble with fixed n_e. The partition function is

$$Z = \sum_i g_i e^{-E_i/k_B T}. \tag{S18}$$

For our simple modeling in Figure 3e of the main text, we consider only the S and P single-carrier levels. Each microstate i is therefore defined by the number of S electrons $n_{e,S,i}$ and number of P electrons $n_{e,P,i} = n_e - n_{e,S,i}$. The energy of microstate i is

$$E_i = n_{e,P,i} \Delta E_e, \tag{S19}$$

where the S-level energy is defined at zero and ΔE_e is the energy gap between conduction-band S and P levels (Supplementary Note 5). The degeneracy of microstate i is

$$g_i = \binom{g_{e,S}}{n_{e,S,i}} \binom{g_{e,P}}{n_{e,P,i}}, \tag{S20}$$

where $g_{e,S} = 2$ and $g_{e,P} = 6$ are the degeneracies of the electron S and P single-carrier levels, respectively.

The fermion nature of electrons is included in the model by considering only microstates with $n_{e,S,i} \leq g_{e,S}$ and $n_{e,P,i} \leq g_{e,P}$. We obtain the expected number of S and P electrons, for a specific n_e, with

$$\langle n_{e,S} \rangle = \frac{1}{Z} \sum_i n_{e,S,i}\, g_i e^{-E_i/k_B T} \quad \text{and} \quad \langle n_{e,P} \rangle = n_e - \langle n_{e,S} \rangle. \tag{S21}$$

Equivalently, the expected numbers can be obtained by calculating the expected energy of the system and realizing that S electrons have zero energy by definition:

$$\langle n_{e,P} \rangle = \frac{k_B T^2}{\Delta E_e} \frac{\partial \ln Z}{\partial T} \quad \text{and} \quad \langle n_{e,S} \rangle = n_e - \langle n_{e,P} \rangle. \tag{S22}$$

The same procedure is used for the hole occupation of S and P levels for a total number of n_h, but using the valence-band degeneracies $g_{h,S} = 4$ and $g_{h,P} = 12$, representing the combined heavy- and light-hole bands.

S8.2 Comparison of the discrete-microstate model to the continuous Fermi-Dirac model and to a Boltzmann model

In Figure S10, we compare the outcome of the exact microstate model for fermion occupations (equation (S21) or, equivalently, equation (S22)) to the predictions of continuous Fermi–Dirac distribution (equation (S17)). The expected number of S and P electrons are calculated from the continuous Fermi–Dirac distribution by first solving μ from

$$g_S f(0) + g_P f(\Delta E) = n_e, \tag{S23}$$

using $f(E)$ from equation (S17), and then evaluating

$$\langle n_{e,S} \rangle = g_S f(0) \quad \text{and} \quad \langle n_{e,P} \rangle = g_P f(\Delta E). \tag{S24}$$

We see in Figure S10 that the two models produce very similar outcomes. The deviation is 10% (relative) for $n_e = 1$ and $n_e = 2$, and decreases as n_e becomes larger. We accept this this level of deviation for the analysis in Figure 4 of the main text and Supplementary Figure 8a and b, because we need a continuous model to plot the rate constants as a function of n_e. In contrast, we use the more exact discrete model in Figure 3 and 5 of the main text. For completeness, Figure S13 provides a side-by-side comparison of experiment, the continuous Fermi–Dirac model, and the discrete microstate model for the quantum efficiency, total lifetime, and P-state emission fraction.

Figure S10 also shows the outcome of a simple Boltzmann model, according to which

$$\langle n_{e,S} \rangle = \frac{g_S}{g_S + g_P e^{-\Delta E / k_B T}} \quad \text{and} \quad \langle n_{e,P} \rangle = n_e - \langle n_{e,S} \rangle. \tag{S25}$$

This model does not account for the fermion nature of electrons. Nevertheless, we see that the Boltzmann model reproduces the discrete model exactly for $n_e = 1$. This is intuitive, as the level occupations are in no way restricted by Pauli exclusion for $n_e = 1$. Indeed, only two microstates are possible for $n_e = 1$ (the electron occupies either an S or a P level) and the microstate degeneracies of equation (S20) reduce to the single-carrier level degeneracies. We thereby obtain equation (S25) exactly.

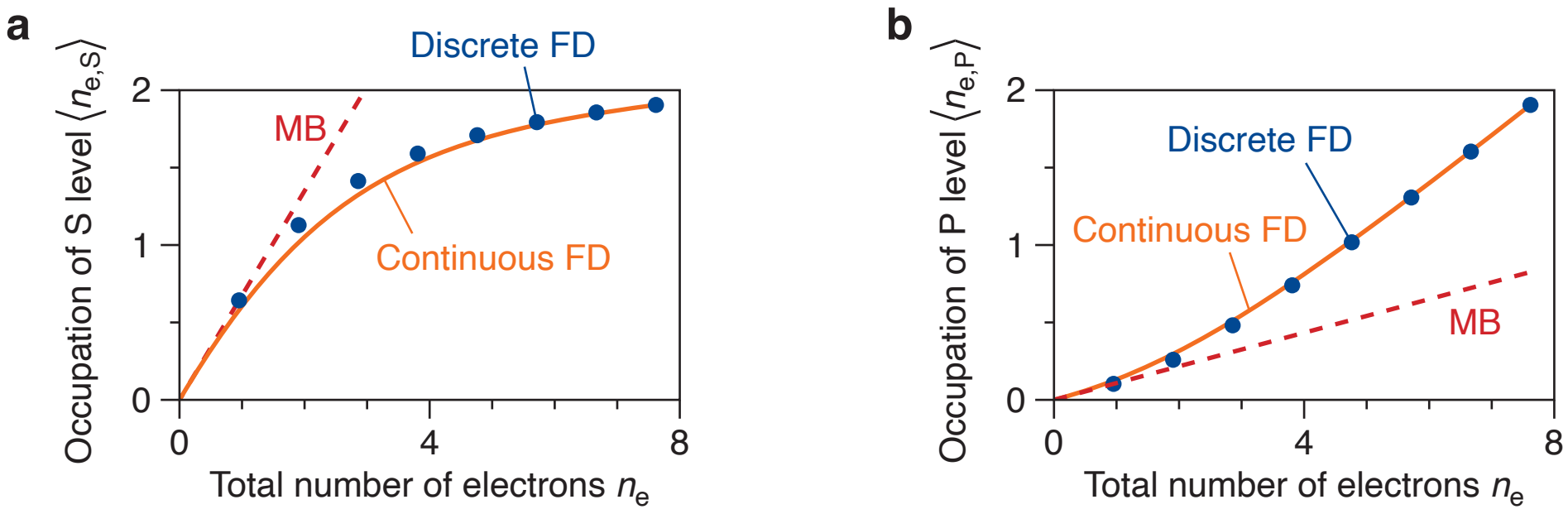

Figure S10 | Level occupations according to different models. **(a)** The calculated occupation of the electron S level $\langle n_{e,S} \rangle$ as a function of the total number of electrons n_e in a microstate, for an S–P energy gap of $\Delta E = 47$ meV. Blue datapoints: discrete Fermi–Dirac model (equation S21). Orange line: the well-known continuous Fermi–Dirac model (equations S17, S24). Red dashed line: the Boltzmann model (equation S25). **(b)** Same as **a**, but for the occupation of the electron P level $\langle n_{e,P} \rangle$.

S8.3 Radiative recombination rates

Radiative recombination occurs between electron–hole pairs occupying levels of the same symmetry (S–S, P_x–P_x, etc.). The emission rate from each channel is therefore estimated as:

$$k_{rad}^{SS} = B' n_e^S n_h^S, \qquad k_{rad}^{PP} = \tfrac{1}{3} B' n_e^P n_h^P, \tag{S26}$$

with the total rate

$$k_{rad} = k_{rad}^{SS} + k_{rad}^{PP}. \tag{S27}$$

Here B' is a universal scaling factor—which is equal for the SS and PP transitions if the oscillator strengths are equal—and the additional factor of 1/3 for the P-state recombination accounts for the requirement of matching orbital orientation (Supplementary Note 5.2).

Equation (S26) is applied to both models: (i) the continuous Fermi–Dirac model (Figure 4e–g, main text), where $\langle n_{e,h}^{S,P} \rangle$ are continuous average occupations, and (ii) the discrete microstate model (Figure 5, main text), where $\langle n_{e,h}^{S,P} \rangle$ are obtained from an average over microstates with fixed integer carrier numbers. In both cases, a single value of B' is chosen such that the calculated total radiative decay rate of the uncharged biexciton matches the experimental value.

The hole degeneracies are relatively large, so the fermionic restrictions play only a minor role. As a result, the number of holes in the S and P levels for $n_h = 2$ is approximately twice that for $n_h = 1$ (to within 4% for S and 2% for P). We therefore divide the experimental $k_{rad,S}$ and $k_{rad,P}$ by the number of holes n_h to collapse the data onto a single curve in Figure 4f–g of the main text.

S8.4 Auger recombination and quantum efficiency

To analyse non-radiative recombination, we assume that the dominant processes are Auger recombination through the positive and negative trion pathways. The prefactors C^+ and C^- were obtained by fitting Eq. 9 of the main text to the experimental non-radiative decay rates. The values were then used to extrapolate Auger recombination rates to higher multi-carrier states up to $n_e = 5$ and $n_h = 5$.

The quantum efficiency is calculated as

$$Q = \frac{k_{\mathrm{rad}}}{k_{\mathrm{Auger}} + k_{\mathrm{rad}}}, \tag{S28}$$

and the total recombination rate as

$$k_{\mathrm{tot}} = k_{\mathrm{Auger}} + k_{\mathrm{rad}}, \tag{S29}$$

with the corresponding total lifetime

$$\tau = \frac{1}{k_{\mathrm{tot}}}. \tag{S30}$$

The data provide no indications that the Auger recombination rates could depend on the distribution of electrons and holes over S and P levels. This is why our simple model for Auger recombination rates (Eq. 9 of the main text) depends only on the total number of electrons and holes. In Figure S11, we consider even simpler models, neglecting the difference between the positive- and negative-trion pathways and/or neglecting the discrete nature of the charge-carrier densities in the BNCs. The model of the main text (Eq. 9) is the best match to the experimental data.

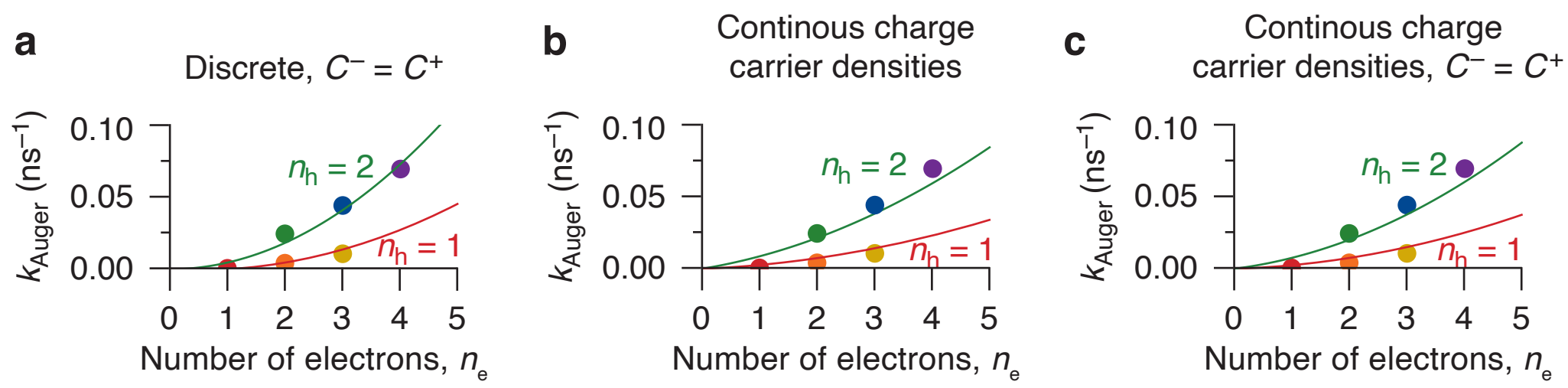

Figure S11 | Alternative Auger models in finite-sized nanocrystals. Total non-radiative decay rate as a function of the number of electrons n_e. Each panel shows the same experimental data as Figure 4h of the main text, but the solid lines are fits to the data using different model assumptions. **(a)** Finite, discrete charge carrier numbers are assumed, with equal rates for the negative-trion and the positive-trion Auger recombination pathways. The model follows $k_{nonrad} = Cn_e n_h(n_e + n_h - 2)$, where the term –2 accounts for the finite number of charge carriers involved. **(b)** Simpler model commonly used for bulk semiconductors that disregards the discrete charge carriers, but with asymmetry in the negative- and positive-trion Auger pathways: $k_{nonrad} = C^- n_e^2 n_h + C^+ n_e n_h^2$. **(c)** Same as **b**, but now assuming equal rates for both Auger pathways: $k_{nonrad} = Cn_e n_h(n_e + n_h)$. The model considered in the main text (Figure 4h; considering finite number of charge carriers and asymmetric positive- and negative-trion Auger pathways) is a better match to the data than any of the models considered here. Specifically, the optimum least-squares loss function of the fit is worse than the for the main-text model by a factor 4 for the model of panel **a**, and by a factor 10 for the models of panels **b** and **c**.

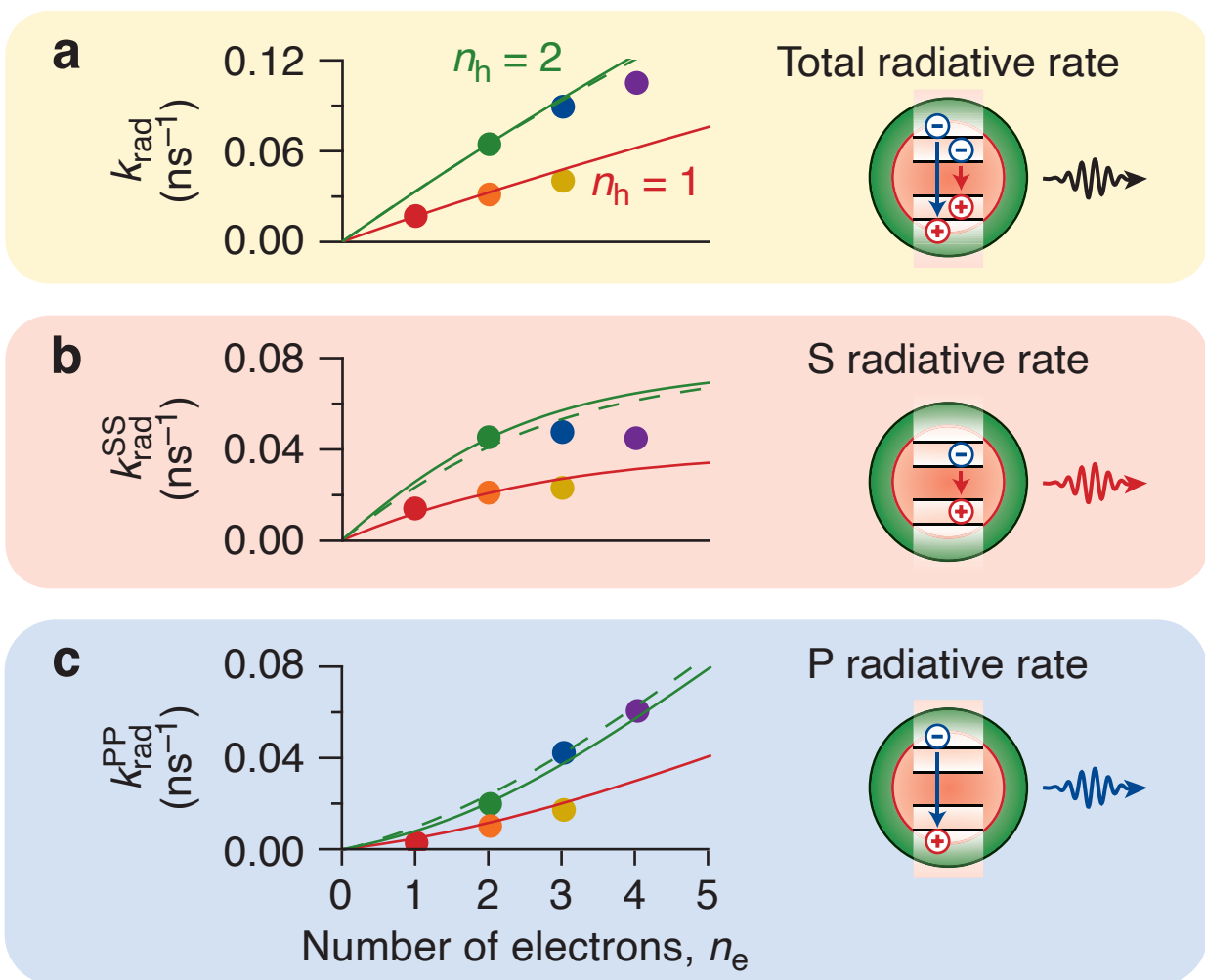

Figure S12 | Model calculations including Coulomb energy shifts in multi-carrier states. Analysis of the peak positions of multi-carrier states (Figure S7) reveals that the S–P energy gap ΔE may be higher by up to 10 meV for (un)charged biexciton states compared to (un)charged exciton states—although the uncertainty is large. **(a–c)** the same type of plots as Figure 4c–e in the main text but redone for a wider energy gap of the biexciton states. Different from the plots in the main text, the *y* values are not normalized by the number of holes n_h in order to emphasize the difference between exciton and biexciton states. Solid lines: ΔE = 57 meV for the states with n_h = 1 hole and 67 meV for states with n_h = 2 holes. Dashed lines: ΔE = 57 meV for all states, as assumed in the main text.

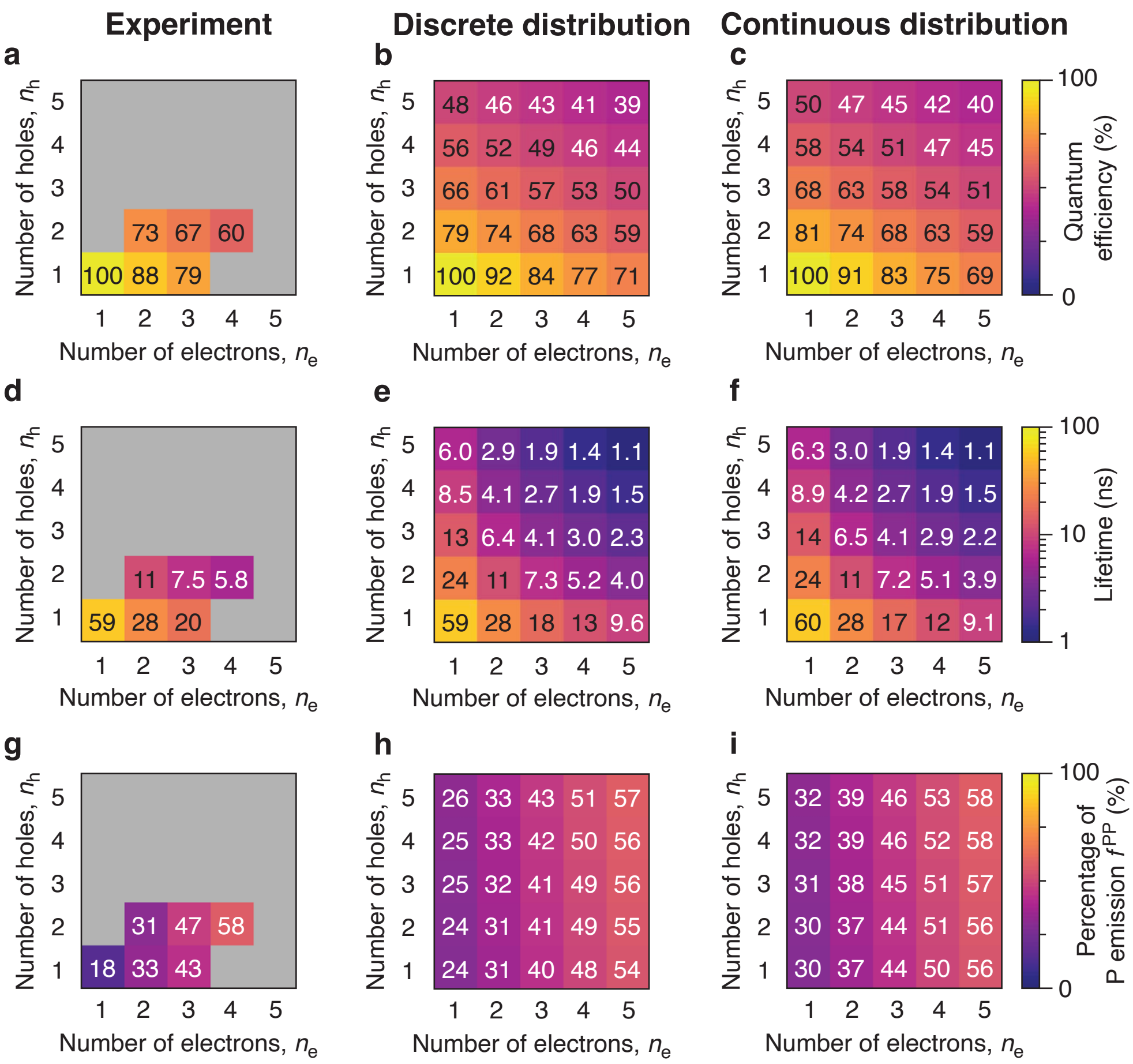

Figure S13 | Comparison between predictions of the continuous and discrete distribution models. (a,d,g) Experimental values of **(a)** quantum efficiency, **(d)** excited-state lifetime, and **(g)** P-state emission fraction, as a function of n_e and n_h for the six multi-carrier states investigated. This is the same data as in Figure 5 of the main text. **(b,e,h)** Corresponding theoretical values using the discrete Fermi–Dirac distribution function (equation S21) and rate constants determined in Figure 4 of the main text. These are the same calculation results as in Figure 5 of the main text. **(c,f,i)** Theoretical values using the continuous but approximate Fermi–Dirac distribution function (equation S17) together with the rate constants of Figure 4 of the main text.

Extended Data

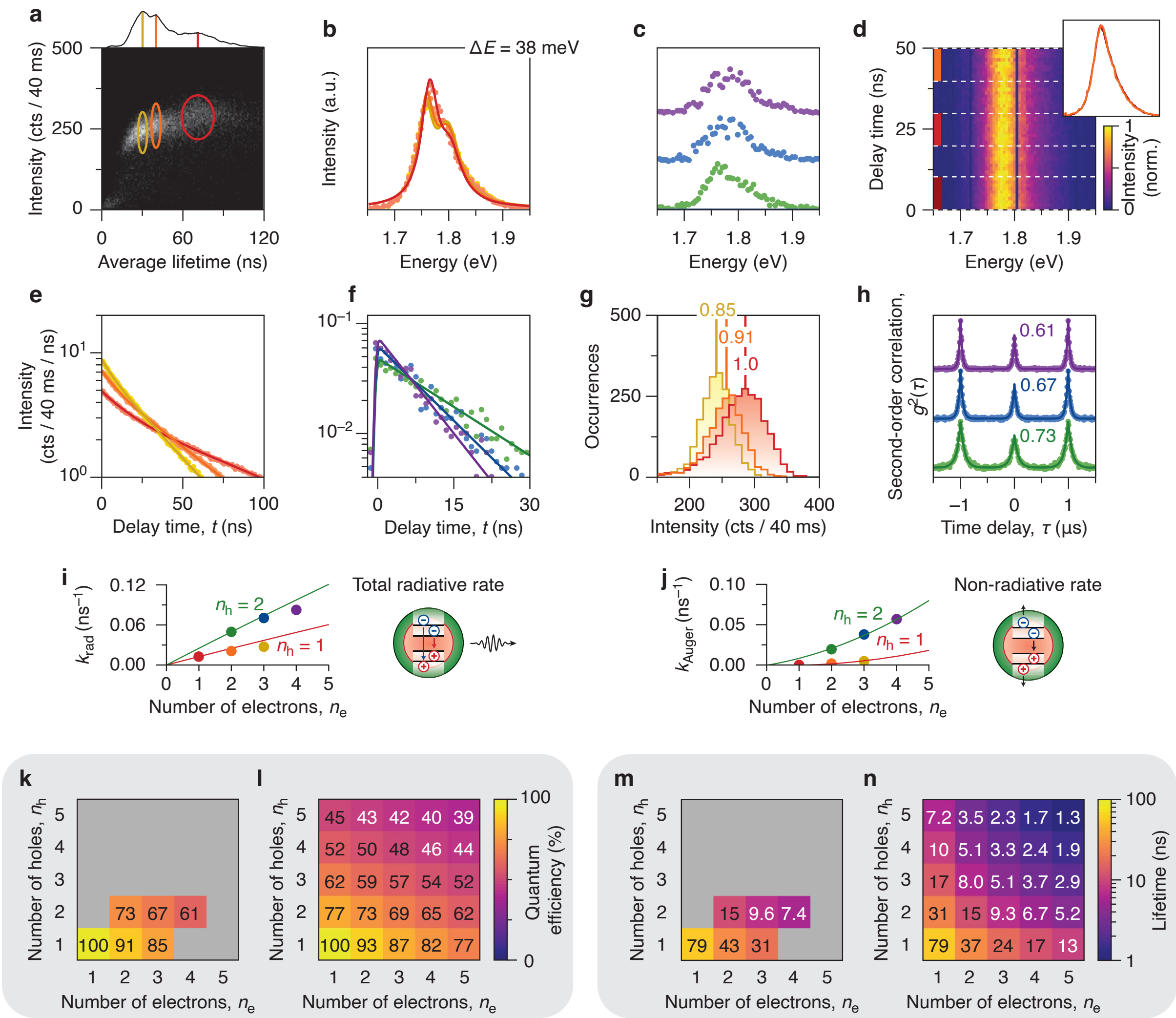

Figure S14 | Summary of the experimental results for a second BNC of the same synthesis batch. **(a)** Fluorescence-lifetime–intensity distribution (FLID), showing fluctuations in average lifetime and intensity across 40-ms time bins, caused by spontaneous charging events in the BNC. Three distinct emissive states are identified. These are indicated in the FLID and in the lifetime histogram (top): neutral (red), singly negatively charged (orange), and doubly negatively charged. **(b)** Reconstructed PL spectra of the neutral exciton (red), singly charged exciton (= trion; orange), and doubly charged exciton (yellow). **(c)** Same as **b**, but for the neutral biexciton (green), singly charged biexciton (blue), and doubly charged biexciton (purple). We observe a rise in the relative intensity of the higher-energy peak with the number of excess electrons, similar to the BNC of the main text. This reflects increased population of the electron P level. **(d)** Normalised time-resolved emission map showing spectral evolution of the neutral exciton emission as a function of decay time. The inset displays the average spectra for delay ranges of 0–10 ns, 20–30 ns, and 40–50 ns. The constant shape of the spectrum over time shows thermal equilibrium between the two emissive states. **(e)** PL decay curves of the neutral (red), singly charged (orange), and doubly charged (yellow) states. Exciton lifetimes are extracted from the slow component of a biexponential fit. **(f)** PL decay curves of the neutral (green), singly charged (blue), and doubly charged (purple) biexciton states, reconstructed by analysing photon cascades. **(g)** Histogram of photon count rates from the neutral (red), singly charged (orange), and doubly charged (yellow) states. The solid lines show the centre of a Gaussian fit. Assuming a quantum efficiency of 1.0 for the neutral state, we estimate the efficiencies of the other states from the relative count rates.[4] **(h)** Second-order correlation functions of the neutral (green), singly charged (blue), and doubly charged (purple) state of the BNC. We estimate the biexciton quantum efficiencies from the peak height in the correlation function and the exciton quantum efficiencies determined in panel **g**. **(i)** The total radiative decay rate as a function of number of electrons n_e. The total radiative rate follows the expected statistical scaling (equation (5) of the main text) for one and two electrons but deviates slightly from the model at higher n_e. We attribute this to a reduced electron–hole wavefunction overlap due to increased electron–electron repulsion at higher occupation. **(j)** The total non-radiative decay rate as a function of n_e. The solid lines represent the expected non-radiative rates for the various charge configurations (equation (9) of the main text) for Auger coefficients $C^- = 0.0009$ and $C^+ = 0.0038$ ns^{-1} for the negative- and positive-trion Auger pathways, respectively. **(k)** Experimental quantum efficiencies of multi-carrier states as a function of the number of electrons n_e and the number of holes n_h. **(l)** Same, but calculated using the model of equations (8) and (9) (of the main text) and using the parameters determined in **i**, **j**. The model is extrapolated to multi-carrier states with up to 5 electrons and 5 holes. **(m–n)** Same as **k,l**, but showing the lifetime of multi-carrier states.

References

1 Cayan, S. A.; Samoli, M.; Tanghe, I.; Lin, C.; Respekta, D.; Hodgkiss, J. M.; Chen, K.; Hens, Z.; Geiregat, P. Stimulated Emission and Lasing from Bulk CdSe Nanocrystals. *J. Phys. Chem. Lett.* **2024**, *15*, 9836–9843.

2 Vonk, Sander J. W.; Rabouw, Freddy T. Biexciton Blinking in CdSe-based Quantum Dots. *J. Phys. Chem. Lett.* **2023**, *14*, 5353–5361.

3 Mangum, B. D.; Ghosh, Y.; Hollingsworth, J. A.; Htoon, H. Disentangling the Effects of Clustering and Multi-Exciton Emission in Second-Order Photon Correlation Experiments. *Opt. Express* **2013**, *21*, 7419–7431.

4 Brokmann, X.; Coolen, L.; Dahan, M.; Hermier, J. P. Measurement of the Radiative and Nonradiative Decay Rates of Single CdSe Nanocrystals through a Controlled Modification of their Spontaneous Emission. *Phys. Rev. Lett.* **2004**, *93*, 107403.

5 Lubin, G.; Yaniv, G..; Kazes, M.; Ulku, A. C.; Antolović, I. M.; Bürri, S.; Bruschini, C.; Charbon, E.; Yallapragada, V. J.; Oron, D. Resolving the Controversy in Biexciton Binding Energy of Cesium Lead Halide Perovskite Nanocrystals through Heralded Single-Particle Spectroscopy. *ACS Nano* **2021**, *15*, 19581–19587.

6 Rech, I.; Ingargiola, A.; Spinelli, R.; Labanca, I.; Marangoni, S.; Ghioni, M.; Cova, S. Optical Crosstalk in Single Photon Avalanche Diode Arrays: a New Complete Model. *Opt. Express* **2008**, *16*, 8381–8394.

7 Maus, M.; Cotlet, M.; Hofkens, J.; Gensch, T.; De Schryver, F. C.; Schaffer, J.; Seidel, C.A. An Experimental Comparison of the Maximum Likelihood Estimation and Nonlinear Least-Squares Fluorescence Lifetime Analysis of Single Molecules. *Anal. Chem.* **2001**, *73*, 2078–2086.

8 Rabouw, F. T.; Kamp, M.; Moes, R.; Gamelin, D.; Koenderink, A.; Meijerink, A.; Vanmakelbergh, D.; Delayed Exciton Emission and its Relation to Blinking in CdSe Quantum Dots. *Nano Lett.* **2015**, *15*, 7718–7725.

9 Reif, F. *Fundamentals of Statistical and Thermal Physics*; McGraw–Hill, **1965**.
